# SnakeAltPromoter Facilitates Differential Alternative Promoter Analysis

**DOI:** 10.34133/csbj.0033

**Published:** 2026-04-09

**Authors:** Jiang Tan, Yuqing Wu, Ruteja Barve, Jared Lalmansingh, Fuhai Li, Philip Payne, Nahyun Kong, Sheng Chih Jin, Yuqi Shan, Ruiwen Zhou, Xinzhou Ge, Jingyi Jessica Li, Richard Head, Yidan Sun

**Affiliations:** ^1^ Department of Genetics, Washington University School of Medicine, St. Louis, MO, USA.; ^2^ Center for Translational Bioinformatics, Washington University School of Medicine, St. Louis, MO, USA.; ^3^ McDonnell Genome Institute, Washington University School of Medicine, St. Louis, MO, USA.; ^4^ Institute for Informatics, Data Science and Biostatistics (I2DB), Washington University School of Medicine, St. Louis, MO, USA.; ^5^ Department of Mathematical Sciences, Carnegie Mellon University, Pittsburgh, PA, USA.; ^6^ Department of Statistics, Oregon State University, Corvallis, OR, USA.; ^7^ Biostatistics Program, Public Health Sciences Division, Fred Hutchinson Cancer Center, Seattle, WA, USA.; ^8^ Department of Biostatistics, University of Washington, Seattle, WA, USA.

## Abstract

**Background:** Alternative promoter usage contributes to isoform diversity and gene regulation in mammals but remains difficult to study at scale. Cap Analysis of Gene Expression precisely maps transcription start sites, but its cost limits large-scale application. Alternatively, ProActiv, Salmon, and DEXSeq can be utilized with widely available RNA sequencing (RNA-seq) data to infer promoter activity. However, there is currently no framework available to automate the generation of reproducible results for these methods. **Results:** SnakeAltPromoter, a scalable end-to-end Snakemake workflow, has been developed to automate alternative promoter analysis from raw RNA-seq data. The workflow performs quality control, alignment, and promoter quantification using 3 complementary RNA-seq analysis methods (junction-based, transcript-based, and first-exon-based), followed by promoter classification and differential activity or usage analysis. SnakeAltPromoter supports both command-line and graphical user interface usage and utilizes standardized modules to enhance reproducibility. A built-in benchmarking module compares promoter activities inferred from RNA-seq data to matched Cap Analysis of Gene Expression data to evaluate quantification performance. Our analyses revealed robust and complementary performance profiles among the evaluated methods across tissues and cell types, highlighting the value of a unified framework for promoter analysis. **Conclusions:** To our knowledge, SnakeAltPromoter is the first unified and reproducible framework that combines scalable execution and guided method selection for RNA-seq-based promoter analysis. By standardizing and integrating existing RNA-seq-based promoter analysis tools, it provides researchers with a robust and accessible platform to investigate promoter-level regulation and alternative promoter activity and will enhance the research value of large, public RNA-seq repositories. Code is freely available at https://github.com/YidanSunResearchLab/SnakeAltPromoter.git.

## Graphical Abstract

**Figure:**
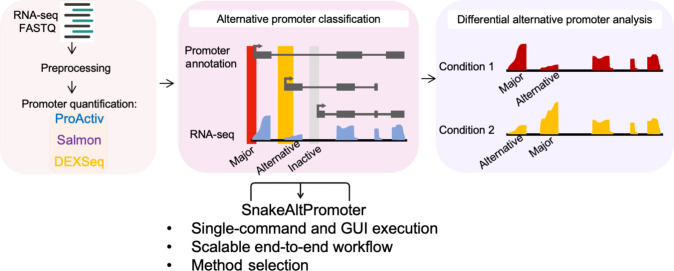

## Introduction

Transcription initiation is regulated by promoters, which integrate inputs from distal regulatory elements and epigenetic modifications [1–3]. In mammals, most protein-coding genes are controlled by multiple promoters, which can drive the expression of distinct isoforms [3–5]. Alternative promoter usage influences isoform diversity through pretranscriptional regulation, rather than posttranscriptional mechanisms, such as alternative splicing [6]. It plays critical roles in diverse biological functions related to development, disease, and cellular reprogramming [7]. Alternative promoters drive organ-specific expression of isoforms during development, enabling precise regulation of processes such as neuronal differentiation [1,5,8]. Aberrant promoter usage in established and novel candidate cancer genes is reported across tissues, cancer types, and patients [9].

Despite the importance of studying alternative promoter usage across biological contexts, substantial barriers remain that limit large-scale studies in this field. Cap Analysis of Gene Expression (CAGE) provides high-resolution transcription start site (TSS) mapping [10] and is well suited for measuring promoter activity, but its cost, technical demands, and limited coverage make it impractical for large cohorts. RNA sequencing (RNA-seq) is a potential low-cost approach to studying alternative promoter usage, and RNA-seq datasets are widely available, but evaluating promoter activity in RNA-seq data accurately remains challenging. A promoter is considered functionally active when RNA polymerase II successfully initiates transcription and progresses into productive elongation, ultimately generating a mature mRNA [11]. Consequently, evaluating alternative promoter activity from short-read RNA-seq is fundamentally different from detecting internal alternative splicing events.

Current strategies for inferring promoter activity from short-read RNA-seq data use state-of-the-art computational strategies to capture different downstream signatures of transcription initiation. These signatures include: (a) junction-based approaches, which quantify reads spanning the first intron as evidence of successful pause-release and commitment to productive elongation (e.g., ProActiv) [9]; (b) transcript-level approaches, which estimate promoter usage through the overall abundance of the resulting mature transcript isoforms (e.g., Salmon) [12]; and (c) exon-based approaches, which measure the specific accumulation of first exons to reflect direct TSS utilization (e.g., DEXSeq) [13]. However, the lack of a community-standard pipeline to guide users in using these strategies and selecting the optimal tool for their specific research is a critical gap that hinders widespread adoption. As a result, most RNA-seq studies do not investigate alternative promoter usage, even when their data could support it.

To address this gap, we developed SnakeAltPromoter, a reproducible and extensible Snakemake workflow for differential alternative promoter analysis from RNA-seq data. SnakeAltPromoter unifies 3 complementary quantification strategies within a single standardized framework that performs promoter classification and both differential activity and usage analyses. The workflow also supports benchmarking against matched CAGE data, enabling systematic comparison of promoter quantification methods to guide optimal method selection. Through its scalable design, streamlined 1-command execution, and graphical user interface, SnakeAltPromoter empowers researchers to generate reproducible results on promoter-level regulation and leverage the wealth of existing RNA-seq data to study transcriptional initiation and isoform diversity.

## Results

### Pipeline overview

**Fig. 1.**
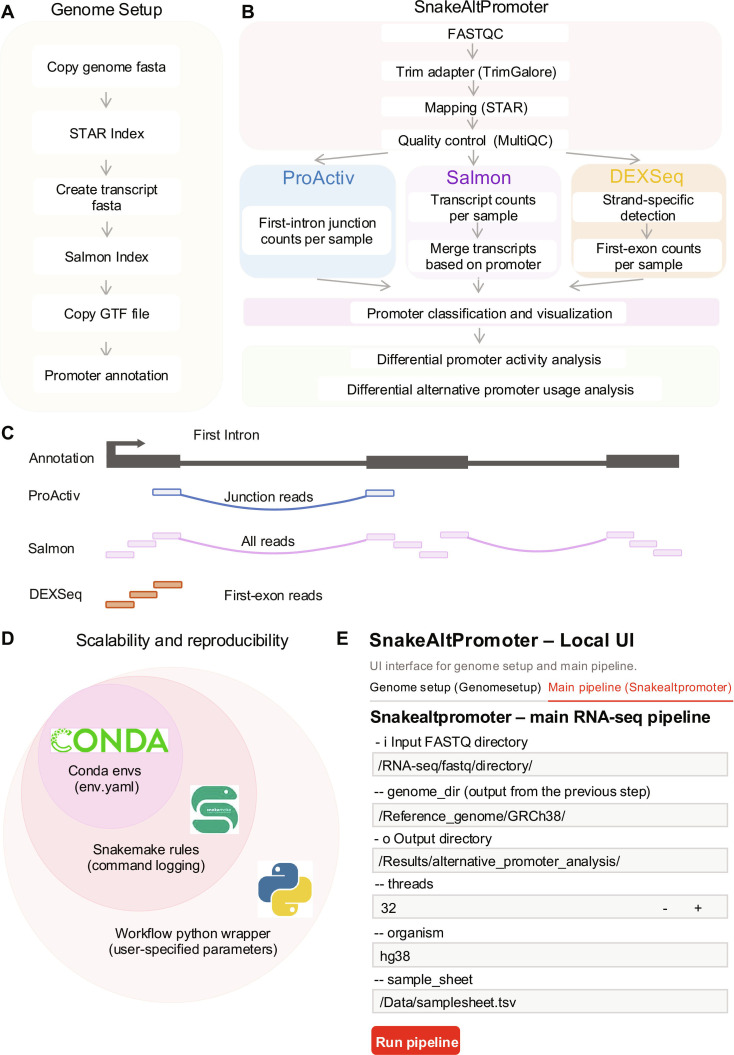
Pipeline overview. Schematic overview of the SnakeAltPromoter pipeline. The workflow is organized into 2 top-level modules: genome setup and alternative promoter analysis. (A) The genome setup module prepares reference files, including genome indices and promoter annotations. (B) The analysis module processes raw FASTQ files through preprocessing, promoter quantification, classification, and differential analysis. (C) Promoter counts are measured using 3 methods: (a) ProActiv, which counts reads from the first-intron junction in STAR junction files, reflecting promoter initiation; (b) Salmon, which quantifies transcript abundances via quasi-mapping and aggregates promoter-level counts using custom R scripts; and (c) DEXSeq/featureCounts, which counts reads in first-exon bins and aggregates them to promoters. (D) Reproducibility is ensured through 3 integrated layers: Conda environments with environment yaml files fix software versions across systems; Snakemake rules record every command, input, and output to ensure traceable execution; and Workflow.py captures user-defined parameters directly from the command line. (E) Streamlit-based graphical user interface provides a user-friendly interface for non-Linux users. The interface allows users to specify input RNA sequencing (RNA-seq) raw fastq directories, genome references, output locations, sample sheets, and computational settings (e.g., threads and organism selection) and execute the pipeline directly through an interactive web interface to improve accessibility for users without extensive command-line experience.

SnakeAltPromoter provides a comprehensive workflow for differential alternative promoter analysis. The workflow is organized into 2 top-level modules: (a) genome setup (Fig. 1A) and (b) alternative promoter analysis (Fig. 1B). The genome setup module is executed once per reference build to generate and store required indices, annotation files, and a unified promoter–transcript–gene map, ensuring consistency across all analyses.

The alternative promoter analysis module processes each RNA-seq project through 4 key steps. Raw FASTQ files first undergo quality control, trimming, and alignment to produce standardized Binary Alignment Maps and quality reports. Promoter activity is subsequently quantified in parallel using 3 complementary strategies that represent distinct computational paradigms for inferring promoter activity (Fig. 1C): junction-based (ProActiv), transcript-based (Salmon), and exon-based (DEXSeq). This ensures comprehensive coverage across gene architectures. Promoters are then classified as major, minor/alternative, or inactive based on their relative expression levels across samples. Finally, differential promoter activity and differential promoter usage analyses are performed using DESeq2 to quantify promoter-level regulatory changes beyond conventional gene-level differential expression.

To enable benchmarking, SnakeAltPromoter can also process matched CAGE data within the same framework, enabling direct comparison of promoter activity inferred from RNA-seq data with CAGE-derived promoter activity profiles. This integrated design provides a unified platform for quantification, classification, differential testing, and performance evaluation across promoter-analysis methods.

### Scalability, reproducibility, and implementation

SnakeAltPromoter efficiently scales across computational environments. On a 32-core workstation, a 50-million-read paired-end human RNA-seq sample completed the full workflow in just over 2 h, with near-linear acceleration when multiple libraries were processed in parallel. The workflow remained memory-efficient (<40 GB) and compute-bound, demonstrating that SnakeAltPromoter can achieve the high throughput required for large-scale promoter analyses.

Reproducibility is ensured through explicit environment management and automated provenance tracking (Fig. 1D). All dependencies and software versions are locked within Conda environment files to ensure consistent execution across systems. Snakemake automatically records every input, parameter, and output and produces an audit trail that allows users to reproduce their results from the same inputs. The built-in Python wrapper for executing Snakemake rules ensures that all parameters are explicitly defined and automatically logged during execution, enabling reproducible analyses without manual configuration files. Example datasets and expected outputs are provided in the GitHub repository to facilitate independent verification.

SnakeAltPromoter is distributed as a standalone tool available via Conda and PyPI and can be executed through a single command line or a Streamlit-based graphical interface. The graphical user interface enables users to upload RNA-seq or CAGE data, select quantification methods (ProActiv, Salmon, or DEXSeq), and adjust statistical thresholds such as false discovery rate (FDR) (Fig. 1E). The combination of these features and Snakemake’s compatibility with common job schedulers (e.g., SLURM) make SnakeAltPromoter accessible for users across computational skill levels and infrastructures.

### Classification of alternative promoters

We applied SnakeAltPromoter to paired RNA-seq and CAGE data generated from human heart tissues isolated from individuals with heart failure and healthy controls [14]. Promoters were defined as regulatory regions upstream of TSSs based on GENCODE v46 annotations [15], yielding 118,403 unique promoters after excluding internal (24,529) and overlapping regions (Table S1).

**Fig. 2.**
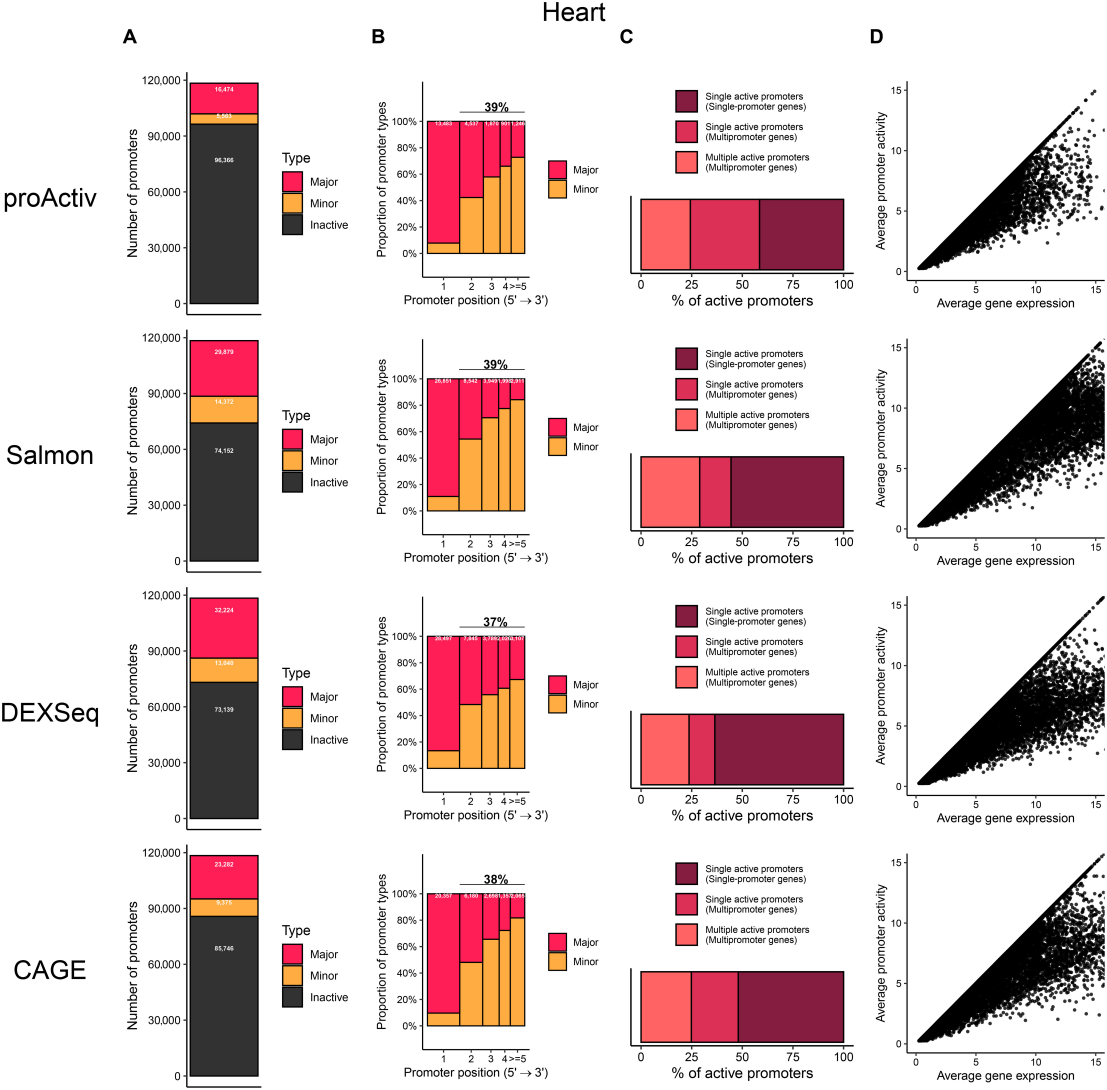
Classification of alternative promoters. (A) Classification of annotated promoters based on average promoter activity across all RNA sequencing (RNA-seq) samples for each method. Promoters are categorized into 3 groups: major promoters, the most active promoter of each gene; minor/alternative promoters, other active promoters of the gene; and inactive promoters, with an estimated activity of <0.25. (B) Distribution of major and minor/alternative promoters across transcription start sites (TSSs), ranked from 5′ to 3′, for multipromoter genes with at least 1 active promoter for each method. (C) Proportions of single-promoter genes with a single active promoter, multipromoter genes with a single active promoter, and multipromoter genes with multiple active promoters for each method. (D) Comparison between major promoter activity and total gene expression (sum of all promoters) for each method. A single promoter often does not fully represent the gene’s expression, as minor/alternative promoters contribute additional regulatory information.

Promoter activity was quantified as log2-transformed counts using ProActiv, Salmon, DEXSeq, and CAGE. Promoters were classified as major (highest activity per gene), minor/alternative (lower activity above a threshold of 0.25), or inactive (no detectable expression) (Fig. 2 and Table S2). Promoter classification results varied slightly between ProActiv (major: 13.91%; minor/alternative: 4.7%; inactive: 81.39%), Salmon (major: 25.23%; minor/alternative: 12.14%; inactive: 62.63%), DEXSeq (major: 27.22%; minor/alternative: 11.01%; inactive: 61.77%), and CAGE (major: 19.66%; minor/alternative: 7.92%; inactive: 72.42%) due to distinct quantification strategies between methods (Fig. 2A). ProActiv [9] relies on junction reads from first introns to identify productive transcription and closely matched CAGE-based initiation signals. ProActiv’s classification of a higher proportion of inactive promoters reflects its limited detection of intronless transcripts. Salmon [12] aggregates transcript abundances, which may dilute promoter-specific signals in long transcripts, while DEXSeq [13] counts first-exon bins, which can include nonproductive transcription.

Empirical data did not always support the assumption that the first annotated promoter is dominant. Major promoters were identified downstream of the first TSS in approximately 37% to 39% of genes (Fig. 2B). Roughly 25% of expressed genes contained multiple active promoters (Fig. 2C and D), highlighting the prevalence of context-dependent promoter usage.

The identification of genes with multiple active promoters in bulk RNA-seq data may reflect both genuine promoter multiplicity and the cellular diversity of heterogeneous heart tissue. To test whether this pattern generalizes, we applied the same analysis to RNA-seq data from human brain tissue and homogeneous cell lines (GM12878 and K562). All datasets showed consistent promoter-usage trends (Figs. S1 to S3), confirming the robustness of these observations across biological contexts.

Collectively, these analyses confirm the prevalence of alternative promoter usage and demonstrate the ability of this workflow to generate regulatory insights from RNA-seq data that are not captured by conventional gene-level expression analysis.

### Benchmarking with CAGE for promoter classification

We used CAGE as an independent reference to evaluate the agreement between RNA-seq-derived promoter classifications and experimentally supported TSSs. CAGE directly captures capped RNA transcripts and is widely used for mapping TSS activity [10]. As CAGE and RNA-seq capture different aspects of transcriptional signals, CAGE serves as a practical reference here rather than a definitive ground truth. Discrepancies between results from the 2 platforms may reflect biological or methodological differences, rather than purely technical noise.

We compared promoter classification in human heart RNA-seq data by ProActiv, Salmon, and DEXSeq against CAGE-derived classifications. We quantified agreement using precision (the fraction of promoters identified by each RNA-seq analysis method that overlap CAGE-defined promoters) and recall (the fraction of CAGE-defined promoters identified by each RNA-seq analysis method).

**Fig. 3.**
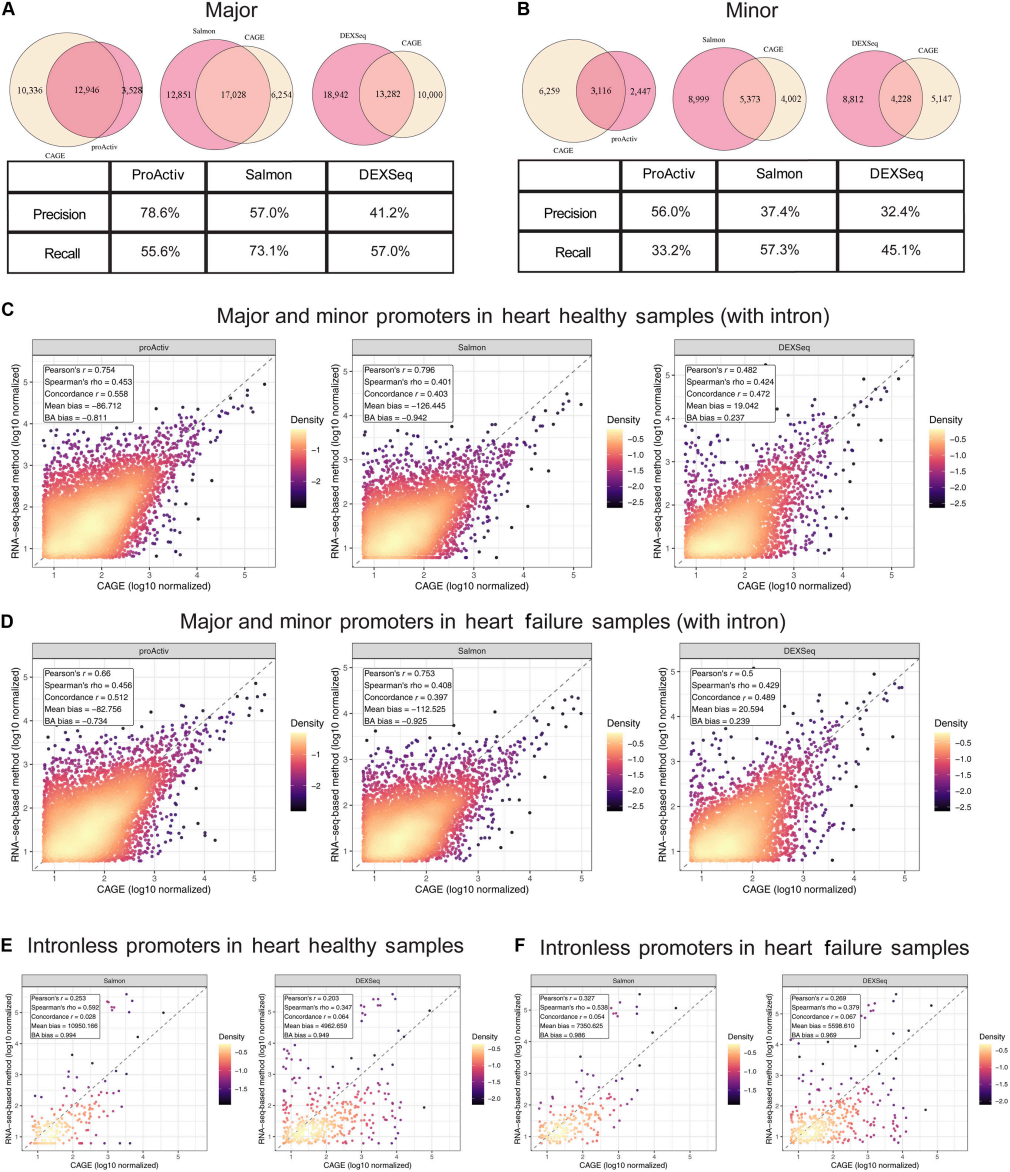
Benchmarking with Cap Analysis of Gene Expression (CAGE) for promoter classification and counts/reads per kilobase per million (RPKM). (A and B) Venn diagrams showing overlap of (A) major promoters and (B) minor/alternative promoters identified by CAGE and each RNA sequencing (RNA-seq) method in all heart samples: ProActiv (left), Salmon (middle), and DEXSeq (right). The overlap represents promoters detected by both the RNA-seq method and CAGE and was used to calculate Precision (Overlap / promoters identified by the RNA-seq method) and Recall (Overlap / CAGE-defined promoters). (C and D) Promoter-wise scatterplots comparing log10-transformed promoter counts/RPKM between CAGE (x-axis) and ProActiv (left), Salmon (middle), and DEXSeq (right) for promoters of intron-containing genes in RNA-seq data from (C) healthy and (D) failed heart tissue; each plot is annotated with the correlation and bias metrics. (E and F) Scatterplots of RPKM of intronless promoters in (E) healthy and (F) failed heart tissue, comparing CAGE to Salmon (left) and DEXSeq (right); plots are annotated with correlation and bias metrics.

ProActiv showed the highest precision (78.6%) for major promoters, reflecting its junction-based design that captures productive transcription near annotated TSSs (Fig. 3A). Salmon and DEXSeq showed lower precision values (57.0% and 41.2%, respectively), as their transcript- and exon-based approaches can also capture signals from nonproductive or paused transcription. Salmon achieved the highest recall of CAGE-defined major promoters (73.1%), followed by DEXSeq (57%), partially because they complement ProActiv’s limited detection of intronless genes. While overall agreement with CAGE was lower for minor or alternative promoters (Fig. 3B), consistent with their weaker expression and coverage, the same pattern held: ProActiv achieved the highest precision relative to CAGE, while Salmon achieved the highest recall.

Collectively, these analyses reveal a characteristic precision–recall trade-off among the evaluated RNA-seq methods. ProActiv achieves the highest precision and strongest agreement with CAGE-supported promoters, consistent with its junction-based design that captures productive transcription initiation, but its limited detection of intronless transcripts results in lower recall. Salmon provides the highest recall, extending promoter detection to intronless loci, but has lower precision relative to CAGE. DEXSeq offers an exon-level framework that captures intronless promoters and shows intermediate behavior in this promoter-specific context. By integrating these complementary strategies, SnakeAltPromoter provides a unified workflow that allows users to balance precision and recall depending on their specific experimental objectives and sequencing configurations.

### Robustness of promoter classification to dataset and technical variation

We performed sensitivity analyses across multiple datasets and technical conditions to evaluate the robustness of promoter classification by each RNA-seq analysis method to both computational and experimental variables.

We conducted benchmarking across RNA-seq datasets from brain tissue and GM12878 and K562 cell lines (Figs. S4 to S6A and B) and found consistent patterns in each RNA-seq analysis method despite dataset-specific variation, supporting the generalizability of these observations.

We evaluated a range of promoter activity thresholds (0.1 to 0.5) to assess the classification stability of each RNA-seq analysis method (Fig. S7A and B). We found that increasing the threshold reduced the total number of active promoters identified, leading to improved precision and decreased recall. Importantly, the relative performance patterns among ProActiv, Salmon, and DEXSeq remained stable across all threshold settings.

We examined the effect of sequencing depth on each RNA-seq analysis method by downsampling heart RNA-seq data from 80 million to 10 million reads (Fig. S7C and D). Increased sequencing depth resulted in higher recall relative to CAGE across all methods, reflecting improved detection of lowly expressed promoters. However, increased sequencing depth also resulted in lower precision relative to CAGE, likely due to increased detection of weak or pervasive transcriptional signals. Despite these changes, the relative performance patterns of ProActiv, Salmon, and DEXSeq remained consistent at sequencing depths above 20 million reads.

Finally, we evaluated the effect of read length on each RNA-seq analysis method using simulated datasets with 50-, 75-, 100-, and 150-bp reads (Fig. S7E and F). Read length had minimal impact on Salmon, consistent with its transcript-level pseudoalignment framework. In contrast, longer reads increased promoter detection by both ProActiv and DEXSeq, resulting in higher recall and slightly reduced precision. ProActiv showed the greatest sensitivity to read length due to its reliance on reads spanning exon–exon junctions, while DEXSeq showed intermediate sensitivity. The relative performance patterns among the 3 RNA-seq analysis methods remained stable across read-length conditions.

These sensitivity analyses demonstrate that promoter classification results are robust across datasets and technical parameters. Although the levels of precision and recall vary with threshold selection, sequencing depth, and read length, the overall performance patterns among ProActiv, Salmon, and DEXSeq remain consistent. These findings support the reliability of promoter classification implemented in the SnakeAltPromoter workflow across diverse RNA-seq datasets and experimental conditions.

### Benchmarking with CAGE for promoter activity quantification

We compared promoter activity outputs from ProActiv, Salmon, and DEXSeq using RNA-seq data from human heart samples to assess quantitative concordance between RNA-seq-derived and CAGE-derived promoter activities (Tables S3 and S5). We normalized Salmon and DEXSeq counts to reads per kilobase per million (RPKM) to improve comparability, while accounting for transcript or exon length differences relative to CAGE initiation signals (Fig. 3C to F). We evaluated agreement using multiple metrics to capture both scale and rank consistency, specifically, Pearson and Spearman correlations, concordance correlation coefficient (CCC), and Bland–Altman bias.

This multimetric benchmarking revealed distinct performance profiles and complementary strengths among the 3 RNA-seq analysis methods across the intron-containing dataset from healthy heart tissue (Fig. 3C). Salmon showed the highest linear correlation with CAGE (Pearson’s *r* ≈ 0.80), supporting its ability to identify relative promoter activity trends and ranking across genes. Salmon showed a stronger negative mean bias (−126.4) and a lower CCC (0.403) relative to CAGE than the other methods, suggesting greater dispersion in magnitude relative to CAGE signals. ProActiv showed a slightly lower correlation than Salmon (Pearson’s *r* = 0.754) but had lower systematic bias (−86.7) and the highest CCC (0.558) of the 3 methods. These results indicate that ProActiv estimates align more closely with the absolute scale of CAGE-derived promoter activity than the other methods, likely due to capturing transcription initiation events more specifically through direct quantification of reads spanning the first intron. DEXSeq showed moderate correlations and CCC overall, reflecting its exon-based statistical framework that captures signals from both productive and promoter-proximal paused transcription. We observed comparable trade-offs between linear correlation and absolute-scale agreement in multimetric benchmarking using the intron-containing dataset from heart failure samples (Fig. 3D), indicating that the performance of the RNA-seq analysis methods is stable across biological conditions.

Intronless promoters are common in the human genome, with 25,627 identified, accounting for 21.65% of all annotated promoters. Genes associated with intronless promoters perform diverse cellular roles across gene ontology (GO) categories, including fundamental cellular functions [16]. Because accurate quantification of these loci is biologically critical, the inability of ProActiv to assess intronless promoters represents a key methodological limitation. To address this, we conducted multimetric benchmarking of only Salmon and DEXSeq using the intronless dataset from healthy and failed heart tissue. Both methods showed reduced concordance with CAGE compared to results for the intron-containing promoter dataset, highlighting the challenge of capturing initiation events without splicing evidence. Salmon showed marginally higher correlations than DEXSeq, supporting its use for the investigation of intronless promoters (Fig. 3E and F).

These results reveal distinct performance profiles among the 3 RNA-seq methods. ProActiv consistently shows strong agreement with CAGE in the quantification of absolute promoter activity levels, while Salmon effectively captures the relative ranking of promoter activities across genes and can quantify intronless promoters that cannot be measured by ProActiv. While DEXSeq is useful for exon-level analyses, it shows moderate concordance in this promoter-specific benchmarking context. Together, this highlights the complementary strengths of each RNA-seq analysis method and provides data to guide method selection for specific promoter analyses within the SnakeAltPromoter workflow.

### Robustness of promoter activity quantification to dataset and technical variation

We conducted the same multimetric benchmarking described above using human brain tissue and GM12878 and K562 cell line datasets to assess the generalizability of our findings in the human heart tissue dataset across biological contexts (Figs. S4 to S6C and D). Although absolute correlation values varied slightly across tissues and cell types, likely due to differences in sequencing depth, transcript complexity, or the quality of available CAGE reference data, the overall performance patterns remained consistent. ProActiv showed the strongest CCC with CAGE across these datasets and had comparable or slightly higher Pearson correlations than Salmon. These results suggest that the relative behavior of the 3 RNA-seq analysis methods is consistent across distinct biological systems.

We examined the influence of sequencing characteristics on promoter activity quantification. We downsampled the heart RNA-seq data from 80 million to 10 million reads and observed that the correlation metrics (Pearson, Spearman, and CCC) for all 3 methods remained remarkably stable across depths ranging from 20 million to 80 million reads. A noticeable drop in correlation occurred at the lowest depth of 10 million reads, indicating that a standard depth of ~20 million reads is sufficient for reliable promoter activity quantification.

We evaluated the effect of read length using simulated datasets with read lengths ranging from 50 to 150 bp (Fig. S8C and D). Salmon showed minimal sensitivity to read-length variation, consistent with its transcript-level quantification strategy. DEXSeq showed a gradual decline in concordance as read length increased. ProActiv showed a slight decrease in Pearson correlation as read length increased, while remaining largely stable across other metrics. This reduction may reflect the increased detection of active promoters with longer reads. Despite these changes in absolute concordance values, the overall performance patterns among the 3 methods remained broadly consistent across read-length conditions.

We performed bootstrap resampling at the promoter level and recalculated Pearson, Spearman, CCC, and Bland–Altman metrics to assess the statistical robustness of the concordance estimates (Fig. S8E). DEXSeq showed greater variability across resampling iterations, while ProActiv and Salmon showed comparatively tighter distributions. This likely reflects the higher sensitivity of DEXSeq’s exon-level counting strategy to local coverage fluctuations. The overall ranking of concordance among methods remained unchanged across bootstrap samples, indicating that the observed performance differences are robust and not driven by a small subset of promoters.

Finally, to evaluate whether benchmarking results were influenced by promoter expression level, we stratified promoters into 6 expression quantiles (Q1 to Q6) based on CAGE activity and recalculated concordance metrics within each stratum (Fig. S8F). As expected, correlations with CAGE remained near zero for low-expression promoters (Q1 to Q3). This likely reflects increased measurement noise, zero-inflation, and sparse signal at low transcript abundance. Concordance began to increase at intermediate levels (Q4 and Q5) especially for Pearson and Spearman correlation and reached its highest values in the top expression bin (Q6). Crucially, the global correlations were consistently lower than those observed in Q6, demonstrating that the overall metrics are not solely driven by highly expressed promoters but instead reflect contributions and noise penalties across the full expression spectrum. Furthermore, ProActiv, Salmon, and DEXSeq exhibited highly similar, nearly parallel trends across expression strata. This indicates that the comparative benchmarking conclusions regarding method behavior remain robust and consistent, primarily reflecting the underlying signal-to-noise characteristics of the data rather than method-specific artifacts.

Together, these analyses demonstrate that the comparative performance of ProActiv, Salmon, and DEXSeq is robust across biological datasets and technical sequencing parameters. Although absolute concordance varies with sequencing depth, read length, and promoter expression level, the overall method-specific performance patterns remain stable. Our results suggest that a sequencing depth of ~20 million reads is sufficient for reliable promoter activity quantification, while read-length effects are modest, with ProActiv and DEXSeq showing slight decreases in concordance at longer reads. These findings support the general applicability of the SnakeAltPromoter workflow across diverse RNA-seq datasets and experimental settings.

### Benchmarking with CAGE for differential promoter activity analysis

**Fig. 4.**
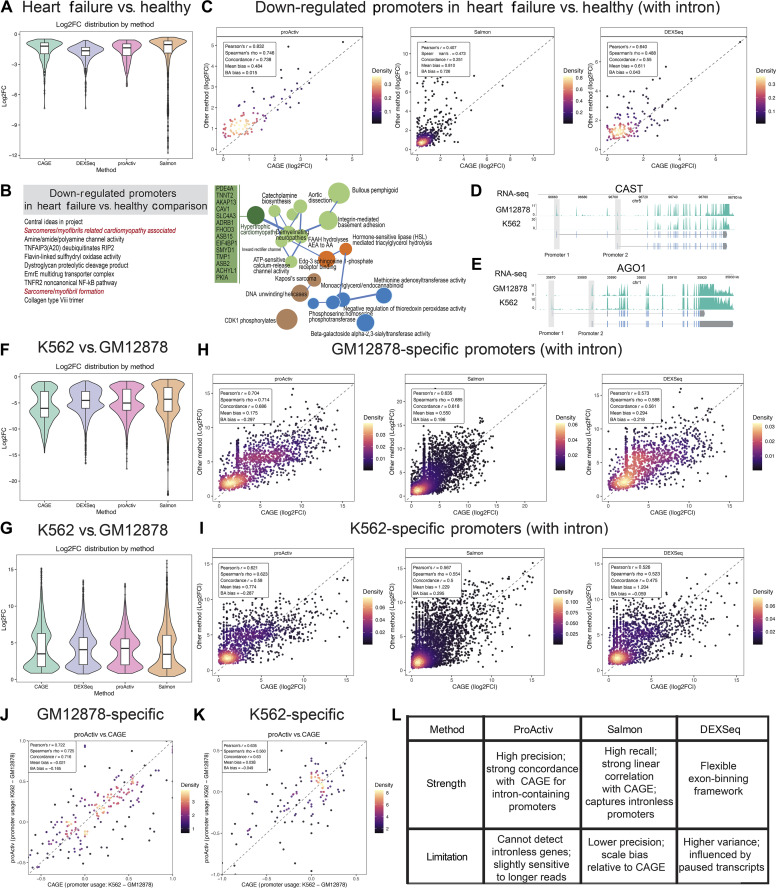
Benchmarking with Cap Analysis of Gene Expression (CAGE) for differential alternative promoter analysis. (A) Violin plot showing log_2_ fold changes (log2FC) of down-regulated promoters between healthy and failed heart samples as detected by ProActiv, Salmon, DEXSeq, and CAGE (false discovery rate [FDR] < 0.05). (B) Pathway map of down-regulated promoters in failed heart samples, as compared to healthy controls, using the CompBIo (v2.9) platform. The resulting map illustrates key pathways and processes associated with heart failure. Prominent themes include hypertrophic cardiomyopathy, sarcomere/myofibril organization, signal conduction, and axon/myelin maintenance. Pathways are color-coded as follows: signal conduction (green), metabolic processes (blue), DNA and nuclear processes (brown), and lipid metabolism (orange). The hypertrophic cardiomyopathy theme (dark green) is highlighted, along with its top enriched genes. (C) Promoter-wise scatterplots comparing log2FC of down-regulated promoters of intron-containing genes in failed heart tissue, as compared to healthy controls, between CAGE (x-axis) and ProActiv, Salmon, and DEXSeq (y-axis); each panel is annotated with the correlation and bias metrics. (D and E) Representative examples of alternative promoter usage. In the *CAST* locus, the upstream promoter is preferentially active in GM12878 cells, and the downstream promoter is preferentially active in K562 cells, as evidenced by the RNA sequencing (RNA-seq) coverage profiles. Conversely, in the *AGO1* locus, the downstream promoter is preferentially active in GM12878 cells, and the upstream promoter is preferentially active in K562 cells. (F) Violin plot showing log2FC of GM12878-specific promoters, which are down-regulated promoters (FDR < 0.05) identified by ProActiv, Salmon, DEXSeq, and CAGE in the K562 versus GM12878 comparison. (G) Violin plot showing log2FC of K562-specific promoters, which are up-regulated promoters (FDR < 0.05) detected by each method and CAGE in the K562 versus GM12878 comparison. (H) Scatterplots of log2FC for GM12878-specific promoters of intron-containing genes (down-regulated in K562 versus GM12878). CAGE (x-axis) versus ProActiv, Salmon, and DEXSeq (y-axis); each panel is annotated with the correlation and bias metrics. (I) Scatterplots of log2FC for K562-specific promoters of intron-containing genes (up-regulated in K562 versus GM12878). CAGE (x-axis) versus ProActiv, Salmon, and DEXSeq (y-axis); each panel is annotated with the correlation and bias metrics. (J) Scatterplot comparing usage shifts in GM12878-specific promoters of intron-containing genes between GM12878 and K562 as measured by CAGE (x-axis) and ProActiv (y-axis); each panel is annotated with the correlation and bias metrics. (K) Scatterplot comparing usage shifts in K562-specific promoters of intron-containing genes between GM12878 and K562 as measured by CAGE (x-axis) and ProActiv (y-axis); each panel is annotated with the correlation and bias metrics. (L) Summary of observed strengths and limitations of the evaluated RNA-seq-based promoter quantification methods based on the benchmarking analyses presented in this study.

We compared differential promoter activity outputs from ProActiv, Salmon, and DEXSeq using RNA-seq data from healthy and failed human heart tissue to benchmark each method to CAGE-derived differential promoter activity. We conducted differential analysis using DESeq2 on promoter counts from CAGE, ProActiv, Salmon, and DEXSeq (Table S4) and found widespread loss of promoter activity in failed heart tissue, most prominently in the CAGE dataset (Fig. 4A).

We conducted GO enrichment analysis on the down-regulated promoters identified by ProActiv, Salmon, and DEXSeq and found enrichment of the following GO terms: “Striated Muscle Contraction”, “Negative Regulation of Cation Transmembrane Transport”, “Regulation of Heart Contraction”, “Cardiac Muscle Contraction”, and “Cardiac Muscle Cell Action Potential”. These terms align with well-established features of heart failure, supporting the relevance of promoter-level signatures captured by SnakeAltPromoter (Fig. S9A). Consistent with these enrichment results, pathway analysis of down-regulated promoters showed coordinated repression of genes involved in cardiac structure and contractility, including sarcomere organization, signal conduction, and myofibril assembly. The most significantly down-regulated genes included hypertrophic cardiomyopathy-related genes, such as PDE4A, TNNT2, AKAP13, CAV1, and FHOD3 (Fig. 4B). This suggests promoter-level repression of key pathways underlying adverse cardiac remodeling.

We compared log₂ fold changes (log₂FC) in the activity of promoters of intron-containing genes from ProActiv, Salmon, and DEXSeq to CAGE in healthy and failed hearts (Table S5). Across all metrics, ProActiv showed the strongest overall agreement with CAGE, achieving the highest linear (Pearson’s *r* = 0.83) and rank-based (Spearman’s ρ = 0.75) correlations and the highest CCC (0.74). ProActiv showed low mean bias (0.48) and minimal Bland–Altman trend (0.015), indicating both proportional and directional consistency with CAGE (Fig. 4C). DEXSeq also showed strong correlations with CAGE, while Salmon showed comparatively lower concordance. These results demonstrate that junction-based quantification not only aligns with CAGE in static promoter classification but also captures dynamic changes in promoter activity during disease.

We compared matched RNA-seq and CAGE data from GM12878 and K562 cell lines to evaluate the performance of RNA-seq analysis methods across biological contexts. We identified up-regulated promoters as K562-specific promoters and down-regulated genes as GM12878-specific promoters by comparing K562 to GM12878 (Fig. 4D to G). Figure 4D and E illustrates reciprocal promoter usage at 2 representative loci, *CAST* and *AGO1*, where the upstream promoter is preferentially active in GM12878 and the downstream promoter in K562 (Fig. 4D) or vice versa (Fig. 4E). The overall performance ranking of each RNA-seq analysis method was consistent with that observed in heart tissue. ProActiv exhibited the highest overall concordance with CAGE across all metrics, followed by Salmon and DEXSeq across most metrics (Fig. 4H and I). Both Salmon and DEXSeq showed substantially lower concordance with CAGE for intronless promoters across all correlation and agreement metrics (Table S5). Correlations were generally weak (most <0.5) with pronounced bias and variability (Fig. S10A to C), indicating systematic deviations in both fold change magnitude and direction. These results highlight the inherent difficulty of inferring the activity of intronless promoters from RNA-seq data.

We compared promoter usage changes inferred by each RNA-seq analysis method to CAGE-derived outputs in GM12878 and K562 cell lines to further assess each method’s ability to detect alternative promoter usage. ProActiv showed the highest overall concordance with CAGE in both cell types across all metrics (Table S5), with Pearson’s *r* = 0.72 and 0.64, Spearman’s ρ = 0.72 and 0.56, and CCC = 0.72 and 0.63 for GM12878- and K562-specific promoters, respectively (Fig. 4J and K and Fig. S10D and E). Bias and Bland–Altman trend values were minimal, indicating limited systematic deviation. Salmon and DEXSeq showed lower correlations with CAGE and higher bias, reflecting less precise estimation of promoter usage changes. These findings validate ProActiv’s robustness in capturing context-specific promoter usage dynamics from RNA-seq data, closely mirroring promoter activity shifts measured by CAGE.

Overall, similar performance patterns were observed across tissues and cell types, with ProActiv generally showing higher concordance with CAGE in differential promoter activity and usage analyses. Salmon and DEXSeq provide complementary coverage, particularly for intronless genes, although they tend to show lower concordance in this differential analysis context. These results highlight the complementary behavior of existing RNA-seq-based promoter quantification strategies when evaluated within a unified analytical framework.

## Methods

### Pipeline architecture

SnakeAltPromoter is implemented in Snakemake (v9.11.2) [17], providing a readable, scalable workflow that runs on local workstations, high-performance computing clusters, and cloud platforms. All third-party software is distributed through Conda/Bioconda [18], which eliminates manual dependency management and the need for administrator privileges.

### Genome setup

We downloaded the primary FASTA and comprehensive GTF for GRCh38 (GENCODE v46 [15]). We indexed the FASTA for STAR (v2.7.11b) [19] and Salmon (v1.10.3) [12]. We generated promoter coordinates with preparePromoterAnnotation from ProActiv (v1.16.0) [9], merging overlapping first exons and keeping the 5′-most TSS. We restricted the analysis to protein-coding genes, as they have the most reliably annotated transcript structures and promoters.

To avoid ambiguous signal attribution in short-read RNA-seq data, we excluded internal promoters whose TSS overlaps downstream exons of other annotated transcripts. In such cases, reads originating from transcriptional elongation of an upstream promoter can overlap the downstream exon and be misinterpreted as transcription initiation at the internal promoter. Removing these cases improves the reliability of promoter activity estimation.

We reused the resulting promoter-to-transcript-to-gene map in every downstream step to ensure consistent promoter definitions.

SnakeAltPromoter is not restricted to specific organisms. The workflow can be applied to any species provided that appropriate genome FASTA and GTF annotation files are available. Users may supply a list of primary chromosomes to exclude unplaced scaffolds or alternative contigs, which can improve promoter annotation quality.

### Data preprocessing

Raw FASTQ files undergo quality assessment with FastQC (v0.12.1) (https://www.bioinformatics.babraham.ac.uk/projects/fastqc/), followed by adapter and low-quality base trimming (*Q* < 20) using TrimGalore (v0.6.10) (https://github.com/FelixKrueger/TrimGalore). Trimmed reads are aligned to the reference genome with STAR [19] in a 2-pass mode to produce coordinate-sorted BAMs and splice-junction tables.

### Promoter count quantification

SnakeAltPromoter measures promoter counts using 3 separate methods that span the major computational paradigms for promoter quantification: (a) ProActiv (v1.16.0) [9], which counts reads from first-intron junction in STAR junction files, reflecting promoter initiation; (b) Salmon (v1.10.3) [12], which quantifies transcript abundances via quasi-mapping and uses custom R scripts to aggregate promoter-level counts; and (c) DEXSeq (v1.52.0) [13], which counts reads in first-exon bins and aggregates them to promoters.

All 3 methods use the same promoter annotation to ensure comparability. ProActiv and DEXSeq rely on uniquely mapped reads, whereas Salmon incorporates multimapping reads using probabilistic assignment. We selected ProActiv, Salmon, and DEXSeq because they are well established, widely adopted within their respective categories, and supported by extensive benchmarking and community use. Salmon ranks among the top-performing tools for transcript quantification [20]; DEXSeq is a widely used standard for exon-level differential usage analysis, with benchmarking studies showing strong recall among exon-based methods and broad adoption [21]; and ProActiv is one of the few dedicated methods for promoter-level inference and is applied in large pan-cancer and tissue-specific analyses [5,9].

### Definition of major, minor/alternative, and inactive promoters

We categorized promoters into 3 distinct groups (major, minor/alternative, and inactive) based on their log2-transformed counts using getAlternativePromoters from proActiv [9]. The promoter with the highest average activity for each gene across all RNA-seq samples was designated as the major promoter. Promoters with an average activity below 0.25 were classified as inactive, while the remaining promoters with intermediate activity were categorized as minor/alternative promoters. The threshold for inactive promoters was the default threshold (minAbs = 0.25) defined in the ProActiv package and described by Demircioğlu et al. [9]. We retained the same criterion here to ensure consistency. We performed a sensitivity analysis using multiple activity thresholds (0.1 to 0.5) to evaluate the robustness of promoter classification to threshold selection.

### Differential promoter activity analysis

For each quantification method, we applied DESeq2 (v1.46.0) [22] to identify differentially expressed promoters between 2 groups (e.g., control versus disease) using an FDR cutoff of 0.05. As our design involved only 2 conditions, we used the default Wald test in DESeq2 to estimate and assess log₂ fold changes between groups. We modeled biological replicates (2 to 3) for each condition explicitly in the DESeq2 design formula and included covariates such as condition and sample type when applicable. When technical or batch effects were present, we incorporated them into the DESeq2 design (e.g., ~ batch + condition) to control for unwanted variation across sequencing runs or experimental batches. Prior to statistical testing, we conducted a goodness-of-fit sanity check to assess replicate concordance, dispersion trends, and outlier samples, ensuring data quality and model validity. We performed library size normalization using DESeq2’s median-of-ratios method and applied variance shrinkage of log₂ fold changes using the empirical Bayes shrinkage (lfcShrink) function to stabilize estimates for promoters with low read counts. We performed multiple testing correction using the Benjamini–Hochberg procedure (FDR < 0.05).

### Differential alternative promoter usage analysis

For each quantification method, we calculated promoter usage as the ratio of individual promoter counts to total gene expression counts. We calculated promoter usage shifts by subtracting promoter usage between 2 conditions for each differentially expressed promoter identified previously. Following the strategy used in the original human heart study [14], we retained promoters showing an absolute usage shift greater than 0.1. We adopted this effect-size threshold to facilitate comparison with previous studies and to identify biologically meaningful promoter usage changes with limited replicate numbers. In more controlled experimental settings, users may additionally apply more strict effect-size filtering.

For statistical testing, we analyzed promoter-level count tables generated by ProActiv, Salmon, or DEXSeq using the getAlternativePromoters framework from the ProActiv package. This framework models promoter-level counts using DESeq2, which applies a negative binomial distribution to account for biological and technical variability. The method evaluates both absolute promoter activity and promoter usage across conditions. Alternative promoter usage is defined when both measures show significant changes simultaneously. The framework constrains gene-level expression fold change to ensure that the detected usage changes represent true compositional shifts, rather than artifacts of large global gene expression changes.

### CAGE data processing

We conducted an identical preprocessing and alignment workflow for raw CAGE FASTQ files as was used for RNA-seq. Briefly, FastQC evaluates read quality, TrimGalore removes adapters and low-quality bases, and STAR [19] is run in a 2-pass mode to align reads to the reference genome.

To enable coordinate-matched benchmarking with RNA-seq-derived promoter activity estimates, we did not apply de novo TSS clustering pipelines (e.g., CAGEr). Instead, we quantified CAGE signals using featureCounts (Subread v2.1.1) directly within the same predefined promoter intervals derived from the reference GTF annotation used for RNA-seq quantification.

We processed the resulting promoter-by-sample count matrix through the same promoter classification and differential analysis modules: Promoters were labeled as major, minor/alternative, or inactive based on log2-transformed counts, and DESeq2 [22] was used to identify differentially active promoters (FDR < 0.05). We calculated promoter usage and usage shifts using the same method described above. This consistent treatment ensures that CAGE data integrates seamlessly into all downstream promoter classification and differential promoter analysis comparisons.

### GO enrichment and CompBio pathway analysis

We used genes associated with promoters that were significantly down-regulated in failed versus healthy heart tissue (DESeq2, FDR < 0.05, log₂FC < 0) for functional enrichment analysis. We performed GO enrichment analysis using enrichR (v3.4) [23].

To facilitate biological interpretation, we analyzed the same gene set using CompBio v2.9 (https://becker.wustl.edu/resources/software/compbio/), a literature-based pathway interpretation platform that identifies biological concepts enriched in a gene list using a PubMed-derived knowledge base. CompBio groups related concepts into context-aware biological themes and visualizes their relationships in a pathway map. We performed this analysis using default settings and retained themes meeting the platform’s significance criteria (normalized enrichment score > 1.2 and empirical *P* value < 0.1). We exported the resulting ranked theme table and knowledge map to summarize biological processes associated with differential promoter activity.

### Benchmarking against CAGE

We compared the promoter analysis outputs of ProActiv, Salmon, and DEXSeq to matched CAGE-derived data from healthy and failed human heart tissue samples to evaluate the accuracy of each RNA-seq analysis method. We performed 3 types of comparisons: promoter classification, promoter count estimation, and differential promoter activity.

### Promoter classification comparison

We evaluated the overlap of major and minor/alternative promoters identified by each method with CAGE using VennDiagram (v1.8.2) [24]. We calculated 2 complementary metrics to quantify this agreement, precision and recall.

Precision was defined as the fraction of promoters identified by the RNA-seq analysis method that overlap CAGE-defined promoters:

$$\begin{array}{c} \begin{array}{c} \text{Precision}=(\text{Number of overlapping promoters between the}\;\mathrm{RNA}\\ \;-\mathrm{seq}\;\text{analysis method and CAGE})/\\ (\text{Total number of promoters identified}\;\mathrm{by}\;\text{the}\;\mathrm{RNA}\\ -\mathrm{seq}\;\text{analysis method}) \end{array} \end{array}$$ (1)

Recall was defined as the fraction of CAGE-defined promoters recovered by each RNA-seq analysis method:

$$\begin{array}{c} \begin{array}{c} \text{Recall}=(\text{Number of overlapping promoters between the}\;\mathrm{RNA}\\ \;-\mathrm{seq}\;\text{analysis method and CAGE})/\\ \left(\text{Total number of promoters identified}\;\mathrm{by}\;\text{CAGE}\right) \end{array} \end{array}$$ (2)

We used these metrics to evaluate the concordance between RNA-seq-based promoter classifications and CAGE annotations.

### Promoter counts comparison

We normalized promoter-level counts from Salmon and DEXSeq by transcript or first-exon length, respectively, to compute RPKM values. This approach effectively adjusts for library depth and compositional bias to facilitate comparison across datasets. To visualize concordance, we generated scatterplots of promoter-wise counts or RPKM for each method against CAGE-derived counts using ggplot2 (v3.5.2) [25].

For quantitative assessment, we calculated Pearson and Spearman correlation coefficients, CCC, mean bias, and Bland–Altman bias to evaluate linear concordance, rank concordance, and bias magnitude between RNA-seq-derived and CAGE-derived promoter activity measures. Pearson correlation evaluates linear association, and Spearman correlation assesses rank concordance. CCC measures overall agreement by jointly accounting for correlation and systematic bias between measurements. We used mean bias and Bland–Altman bias to evaluate potential magnitude differences between the 2 platforms. All correlation statistics were computed in R (v4.4.0) using standard or custom functions.

For intronless promoters, which lack first-intron junctions and cannot be quantified by ProActiv, we generated scatterplots of promoter-wise counts for only Salmon and DEXSeq against CAGE-derived counts using ggplot2 [25]. We calculated Spearman correlation coefficients and associated *P* values to account for nonlinear relationships in count data.

### Differential alternative promoter analysis comparison

For differential promoter activity, we processed promoter-level raw counts to compute absolute log2 fold changes (log2FC) in RNA-seq data from healthy and failed heart tissue for each method using DESeq2 [22] (FDR < 0.05) and generated scatterplots [25] against CAGE’s absolute log2FC values. We calculated Pearson and Spearman correlation coefficients, CCC, mean bias, and Bland–Altman bias to evaluate the linear concordance, rank concordance, and magnitude bias of differential promoter activity measures between RNA-seq-derived and CAGE-derived promoter log2FC measures. All statistics were computed in R using standard or custom functions.

To identify cell type-specific promoters, we obtained RNA-seq and CAGE datasets for GM12878 and K562 cell lines from ENCODE. We identified promoters specific to each cell type by comparing their expression between K562 and GM12878 using DESeq2 (FDR < 0.05). We classified promoters as K562-specific if log2FC > 0 or GM12878-specific if log2FC < 0 in K562 versus GM12878 comparisons. We generated scatterplots [25] of log2FC values for these cell type-specific promoters comparing ProActiv, Salmon, and DEXSeq against CAGE, with all metrics calculated to assess concordance in detecting cell type-specific promoter activity.

To detect condition-dependent promoter usage shifts, we first computed promoter usage for each cell type as the ratio of individual promoter counts to total gene expression counts. We then calculated the usage shift by subtracting GM12878 promoter usage from K562 promoter usage for each promoter. We removed promoters with usage shifts <0.1. To evaluate concordance with CAGE, we generated scatterplots [25] of the promoter usage shifts identified by ProActiv against those from CAGE and computed all metrics.

## Discussion

SnakeAltPromoter addresses a critical gap in transcriptomics by providing the first systematic and reproducible framework for alternative promoter analysis from RNA-seq data. Unlike existing tools focused on gene expression or splicing, SnakeAltPromoter targets promoter-level regulation to characterize isoform diversity in a manner that is not possible with standard analyses. The SnakeAltPromoter pipeline unifies ProActiv, Salmon, and DEXSeq RNA-seq analysis methods within a single Snakemake workflow for promoter classification, quantification, and differential analyses and supports optional comparative evaluation against CAGE-derived data. Rather than introducing new quantification algorithms, SnakeAltPromoter advances the field by standardizing and automating existing promoter analysis strategies within a scalable and fully reproducible workflow.

Several design features distinguish SnakeAltPromoter. First, it eliminates coordinate mismatches by using the same harmonized promoter table with all 3 RNA-seq analysis methods. Second, it scales seamlessly from a laptop to an high-performance computing node with near-linear efficiency. Finally, every step, parameter, and software version is automatically tracked through Snakemake and Conda to ensure full provenance and reproducibility.

While the primary scientific advance of this study is the development of the standardized SnakeAltPromoter workflow, we also benchmarked each RNA-seq analysis method systematically against reference CAGE data. By evaluating these tools within a unified framework, our goal is to provide users with an evidence-based guide to understand the respective strengths and limitations of each method, rather than to establish a definitive ranking. Our analyses reveal a clear precision–recall trade-off that is shaped by the underlying methodological designs of each method. ProActiv demonstrates the highest precision and strongest concordance with CAGE for intron-containing promoters, making it particularly reliable for capturing the initiation of productive transcription. However, ProActiv shows sensitivity to read length, and its junction-based design prevents the detection of intronless genes. Salmon’s transcript-level pseudoalignment achieves the highest recall and often strong linear correlations with CAGE, successfully captures intronless loci, and is robust across read lengths but has reduced precision and greater systematic bias relative to CAGE. DEXSeq provides an exon-level framework that is capable of detecting intronless genes, but it exhibits higher statistical variability and moderate concordance to CAGE in this promoter-centric context. By integrating these complementary strategies, SnakeAltPromoter enables researchers to make informed, context-dependent methodological choices to optimize RNA-seq-based promoter analysis for their specific datasets and experimental objectives.

### Expansion to long-read sequencing data

Analysis of short-read RNA-seq data is limited by factors such as ambiguous read mapping at exon junctions or in regions with complex splicing [26,27]. The adaptation of SnakeAltPromoter to long-read sequencing data, such as those generated by PacBio [26] or Oxford Nanopore [27] platforms, is a promising future direction that will address these limitations and provide a robust framework for studying promoter dynamics in complex transcriptomes. ProActiv’s junction-based approach [9] can be extended to long-read data by leveraging full-length transcript alignments to precisely identify TSSs and their associated junctions. As long-read data provide unambiguous evidence of isoforms, this adaptation is likely to enhance accuracy and improve the detection of low-abundance minor/alternative promoters, which is challenging with short-read data [26,27]. Integrating long-read support into SnakeAltPromoter would involve updating the preprocessing module to handle long-read alignment tools (e.g., minimap2 [28]) and modifying ProActiv’s junction detection to process full-transcript data.

Adapting SnakeAltPromoter to identify precise promoter usage from standard long-read RNA-seq data presents several technical challenges. Unlike CAGE or other 5′-cap-capturing approaches, conventional complementary DNA-based long-read sequencing frequently suffers from incomplete 5′ transcript coverage due to RNA degradation or incomplete reverse transcription. As a result, truncated reads mapping downstream of canonical TSSs can be difficult to distinguish from genuine alternative promoters. In addition, the relatively high indel error rates near read termini may complicate precise nucleotide-level TSS localization, particularly for closely spaced promoter clusters. Extending the SnakeAltPromoter framework to long-read data will require the integration of specialized 5′-enriched long-read protocols alongside novel error-correction algorithms specifically calibrated for TSS identification.

### Integration with epigenomic data

Integrating epigenomic data, such as assay for transposase-accessible chromatin using sequencing (ATAC-seq) [29] or chromatin immunoprecipitation sequencing for promoter-associated histone marks (H3K4me3 and H3K27ac), could further enhance SnakeAltPromoter’s capacity to characterize promoter activity. These data provide orthogonal evidence of active TSSs by identifying open chromatin and activating histone modifications that reliably mark promoter regions [29]. Incorporating such datasets would allow the pipeline to validate RNA-seq-derived promoter identification. This is likely to enhance analyses of minor or low-abundance for which RNA-seq analysis methods show reduced concordance with CAGE. For instance, integrating ProActiv’s junction-based counts with ATAC-seq peak intensity measures could improve classification accuracy by confirming promoter activity in low-expression contexts. Implementation would require an additional module that aligns epigenomic reads, calls peaks, and intersects those peaks with the existing promoter atlas. Machine learning models could further combine multiomics data to predict promoter status. Finally, extending the ATAC-seq branch to include digital footprinting (e.g. TOBIAS [30] or HINT-ATAC [31]) would identify transcription-factor binding events within promoter peaks. This would directly link specific transcription factors to promoter usage shifts identified by SnakeAltPromoter and provide mechanistic insight into tissue- or disease-specific regulatory programs.

## Conclusions

SnakeAltPromoter provides a unified, reproducible, and scalable solution for alternative promoter analysis from RNA-seq data. SnakeAltPromoter bridges the gap between algorithm development and practical application by integrating quality control, quantification, classification, and differential testing within a single Snakemake framework. Through systematic benchmarking against CAGE data, SnakeAltPromoter offers an evidence-based guide for selecting the most appropriate quantification strategy across experimental contexts. SnakeAltPromoter establishes a standardized foundation for large-scale and reproducible promoter analysis and enables transparent evaluation and integration of new quantification tools as they emerge. Its modular design and accessibility through both command-line and graphical interfaces make it suitable for diverse research communities regardless of computational expertise. Future developments will extend the framework to long-read and epigenomic datasets and explore integration with epigenetic data to characterize coordinated transcriptional regulation. Together, these capabilities position SnakeAltPromoter as a practical and extensible resource for investigating promoter architecture and transcriptional control across biological systems.

## Ethical Approval

Not applicable.

## Data Availability

The Snakemake pipeline and example data are available at https://github.com/YidanSunResearchLab/SnakeAltPromoter.git. All the RNA-seq and CAGE datasets used in the paper are from Gene Expression Omnibus and European Nucleotide Archive with the accession codes GSE147236, ERP145032, ERP144765, GSE33480, and GSE34448.
