# Supplementary material for: SnakeAltPromoter Facilitates Differential Alternative Promoter Analysis
Source: Comput Struct Biotechnol J. 2026 Apr 9;35(1):0033. doi: 10.34133/csbj.0033 (PMC13082578; doi:10.34133/csbj.0033)
Supplement: Supplementary 1 — Figs. S1 to S10 Tables S1 to S5 [file csbj.0033.f1.zip › Supplemental Figure 4.pdf]

Brain

A

Major

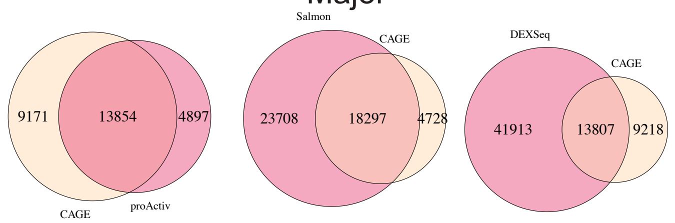

	ProActiv	Salmon	DEXSeq
Precision	73.9%	43.6%	24.8%
Recall	60.2%	79.5%	60.0%

B

Minor

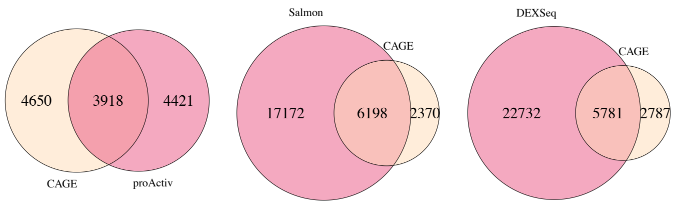

	ProActiv	Salmon	DEXSeq
Precision	47.0%	26.5%	20.3%
Recall	45.7%	72.3%	67.5%

C

Major and Minor Promoters in Brain Samples (with intron)

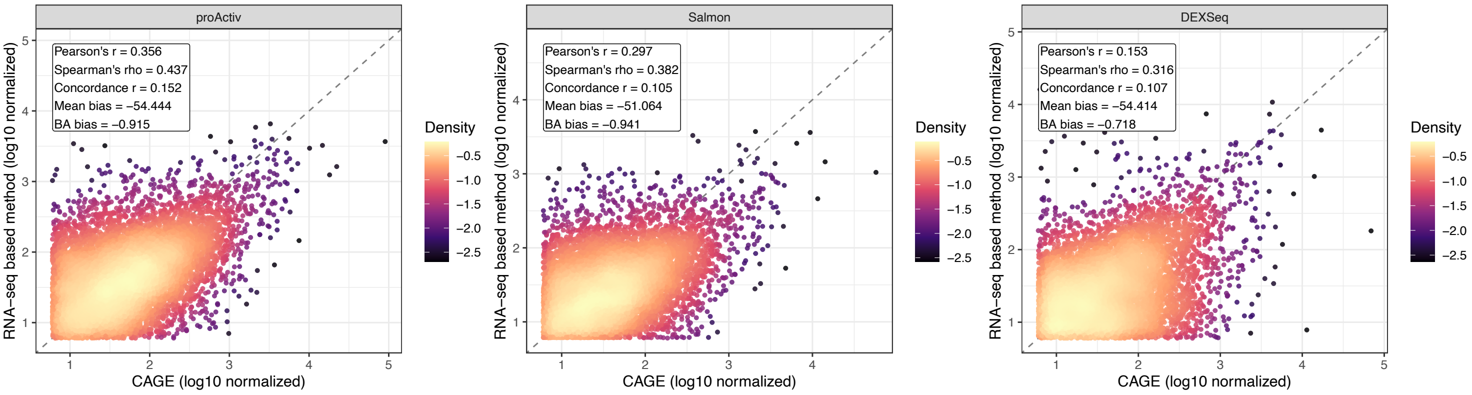

D

Intronless Promoters in Brain Samples

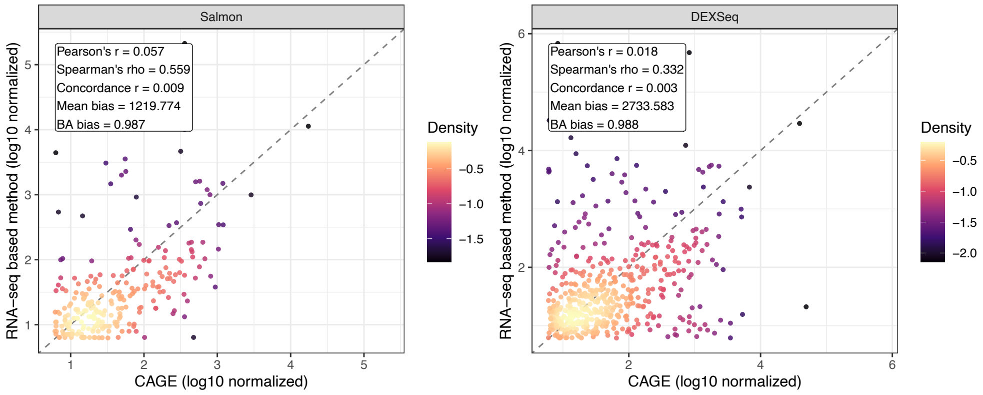

Supplementary Figure 4
