# Supplementary material for: SnakeAltPromoter Facilitates Differential Alternative Promoter Analysis
Source: Comput Struct Biotechnol J. 2026 Apr 9;35(1):0033. doi: 10.34133/csbj.0033 (PMC13082578; doi:10.34133/csbj.0033)
Supplement: Supplementary 1 — Figs. S1 to S10 Tables S1 to S5 [file csbj.0033.f1.zip › Supplementary Legends.docx]

### **Supplementary Files**

**Supplementary Figure 1–3. Classification of alternative promoters in brain tissue and GM12878 and K562 cell lines.**

(A) Classification of annotated promoters based on average promoter activity across all RNA-seq samples for each method. Promoters are categorized into three groups: major promoters, the most active promoter of each gene; minor/alternative promoters, other active promoters of the gene; and inactive promoters, with an estimated activity of < 0.25. (B) Distribution of major and minor/alternative promoters across transcription start sites (TSSs), ranked from 5′ to 3′, for multi-promoter genes with at least one active promoter for each method. (C) Proportions of single promoter genes with a single active promoter, multi-promoter genes with a single active promoter, and multi-promoter genes with multiple active promoters for each method. (D) Comparison between major promoter activity and total gene expression (sum of all promoters) for each RNA-seq analysis method. A single promoter often does not fully represent the gene’s expression, as minor/alternative promoters contribute additional regulatory information.

**Supplementary Figure 4–6. Benchmarking with CAGE for promoter classification and counts in brain tissue and GM12878 and K562 cell lines.**

(A and B) Venn diagrams showing overlap of (A) major promoters and (B) minor/alternative promoters identified by CAGE and each RNA-seq method: ProActiv (left), Salmon (middle), and DEXSeq (right). Precision measures the fraction of RNA-seq–identified promoters supported by CAGE. Recall measures the fraction of CAGE-defined promoters recovered by RNA-seq methods. (C) Promoter-wise scatterplots comparing log10-transformed promoter counts/RPKM between CAGE (x-axis) and ProActiv (left), Salmon (middle), and DEXSeq (right) for promoters of intron-containing genes; each plot is annotated with the correlation and bias metrics. (D) Scatterplots of RPKM of intronless promoters comparing CAGE versus Salmon (left) and DEXSeq (right); plots are annotated with correlation and bias metrics.

**Supplementary Figure 7. Robustness of promoter classification to threshold selection and sequencing parameters.**

(A and B) Effect of promoter activity thresholds on promoter classification. The number of detected promoters, precision, and recall were evaluated across activity thresholds of 0.1, 0.25, and 0.5 for (A) major promoters and (B) minor promoters. Promoter numbers were normalized to the results obtained at threshold = 0.1. (C and D) Effect of sequencing depth on promoter classification. Heart RNA-seq datasets were downsampled from 80 million to 10 million reads and promoter classification performance was evaluated for (C) major promoters and (D) minor promoters. Promoter numbers were normalized to the results obtained at the full dataset (80 million reads). (E and F) Effect of read length on promoter classification using simulated datasets with read lengths of 50, 75, 100, and 150 bp. Results are shown for (E) major promoters and (F) minor promoters. Promoter numbers were normalized to the results obtained at 150 bp read-length condition. Precision measures the fraction of RNA-seq–identified promoters supported by CAGE, recall measures the fraction of CAGE-defined promoters identified by RNA-seq analysis methods.

**Supplementary Figure 8. Robustness of promoter activity to threshold selection and sequencing parameters.**

(A and B) Influence of sequencing depth on promoter activity estimation in the heart dataset. Heart RNA-seq reads were downsampled from 80 million to 10 million reads, and concordance between RNA-seq–derived promoter activities and CAGE was evaluated using Pearson correlation, Spearman correlation, concordance correlation coefficient (CCC), and Bland–Altman bias. Results are shown for (A) healthy and (B) failed heart tissue. (C and D) Influence of read length on promoter activity estimation using simulated RNA-seq datasets with read lengths of 50, 75, 100, and 150 bp. Concordance between RNA-seq–based estimates and CAGE was evaluated using the same metrics for (C) healthy and (D) failed heart tissue. (E) Bootstrap analysis of concordance metrics. Promoters were resampled with replacement and correlation metrics were recalculated to assess statistical stability of the benchmarking results. Distributions of Pearson, Spearman, CCC, and Bland–Altman correlation metrics are shown for healthy (top) and failed (bottom) heart tissue. (F) Influence of promoter expression level on concordance with CAGE. Promoters were stratified into quartiles (Q1–Q6) according to CAGE expression levels and concordance metrics were recalculated within each expression group for healthy (top) and failed (bottom) heart tissue.

**Supplementary Figure 9. Functional enrichment of genes associated with downregulated promoters in heart failure.**

(A) Gene Ontology (GO) biological process enrichment analysis of genes associated with promoters showing reduced activity in failed heart tissue, as compared to healthy controls. Bars represent the combined enrichment score for each GO term, indicating the relative strength of enrichment.

**Supplementary Figure 10. Benchmarking with CAGE for differential promoter analysis.**

(A) Promoter-wise scatterplots comparing log2FC of downregulated intronless promoters failed heart issue, as compared to healthy controls, between CAGE (x-axis) and Salmon or DEXSeq (y-axis). (B) Scatterplots of log2FC for GM12878-specific intronless promoters (downregulated in K562 vs. GM12878) comparing CAGE (x-axis) versus Salmon and DEXSeq (y-axis). (C) Scatterplots of log2FC for K562-specific intronless promoters (upregulated in K562 vs. GM12878) comparing CAGE (x-axis) versus Salmon and DEXSeq (y-axis). (D) Scatterplot comparing usage shifts in GM12878-specific promoters of intron-containing genes as measured by CAGE (x-axis) versus Salmon and DEXSeq (y-axis). (K) Scatterplot comparing usage shifts in K562-specific promoters of intron-containing genes as measured by CAGE (x-axis) versus Salmon and DEXSeq (y-axis). Each panel is annotated with correlation and bias metrics.

**Supplementary Table 1**. A comprehensive list of promoter coordinates.

**Supplementary Table 2**. Classifications of major and minor/alternative promoters using Proactiv, Salmon, DEXseq, and CAGE.

**Supplementary Table 3**. Promoter counts across samples measured by Proactiv, Salmon, DEXseq, and CAGE.

**Supplementary Table 4**. Differential promoter activity analysis between healthy and failed heart tissue measured by Proactiv, Salmon, DEXseq, and CAGE.

**Supplementary Table 5**. Summary of correlation and bias metrics.
