# Supplementary figures and images for: SnakeAltPromoter Facilitates Differential Alternative Promoter Analysis
Source: Comput Struct Biotechnol J. 2026 Apr 9;35(1):0033. doi: 10.34133/csbj.0033 (PMC13082578; doi:10.34133/csbj.0033)

# Brain

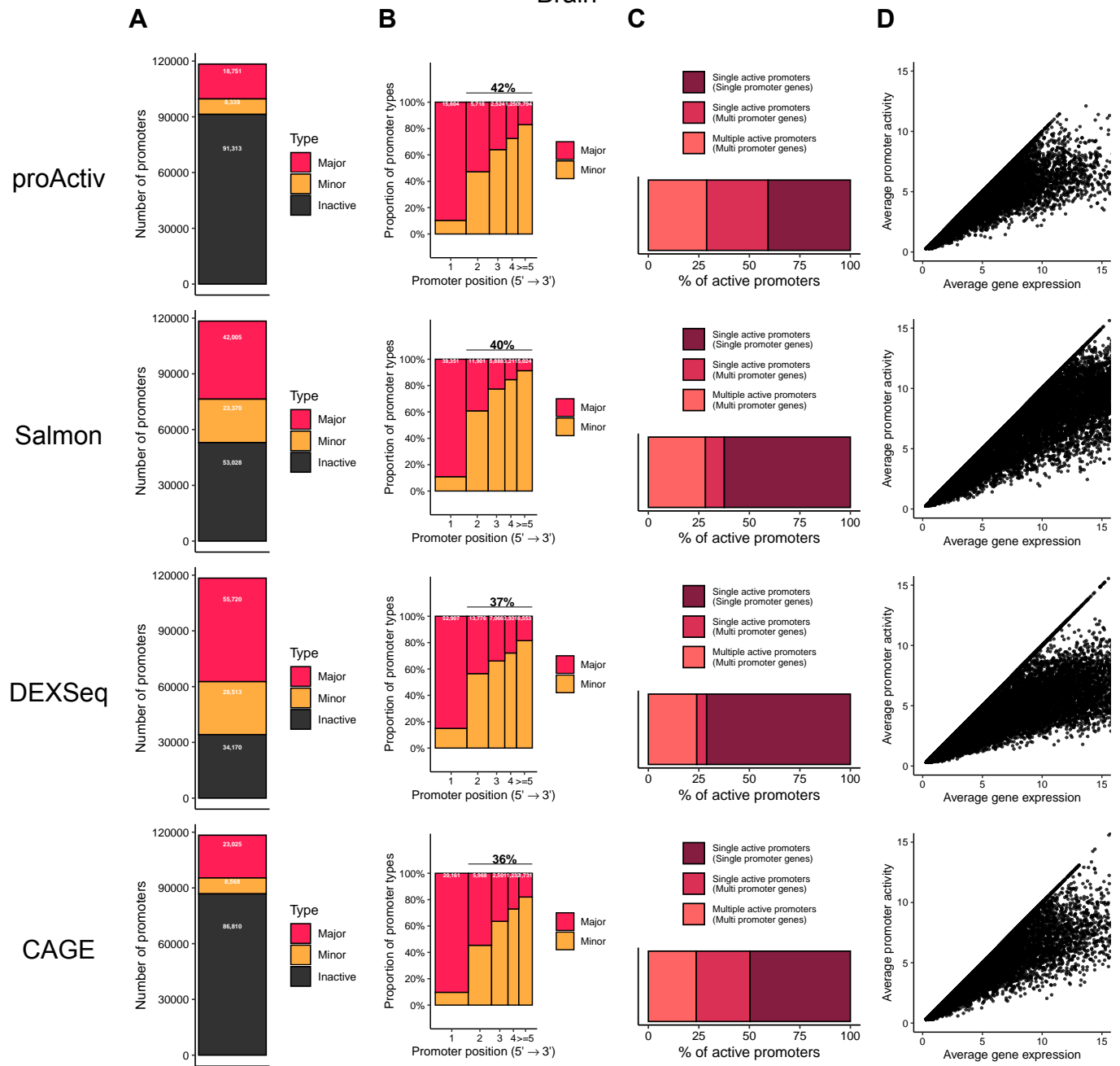

Supplement: Supplementary 1 — Figs. S1 to S10 Tables S1 to S5 [file csbj.0033.f1.zip › Supplemental Figure 1.pdf]

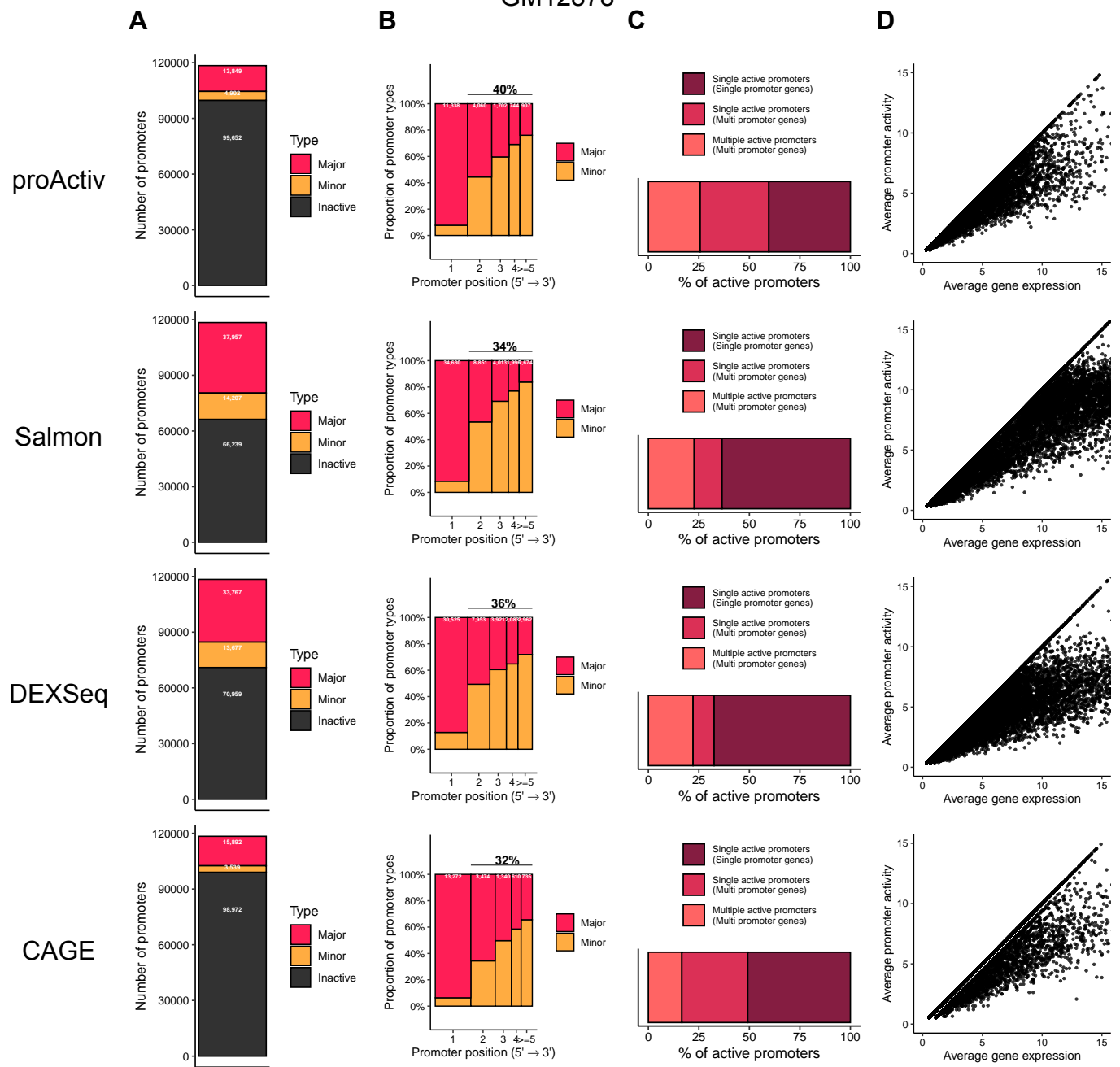

Supplement: Supplementary 1 — Figs. S1 to S10 Tables S1 to S5 [file csbj.0033.f1.zip › Supplemental Figure 2.pdf]

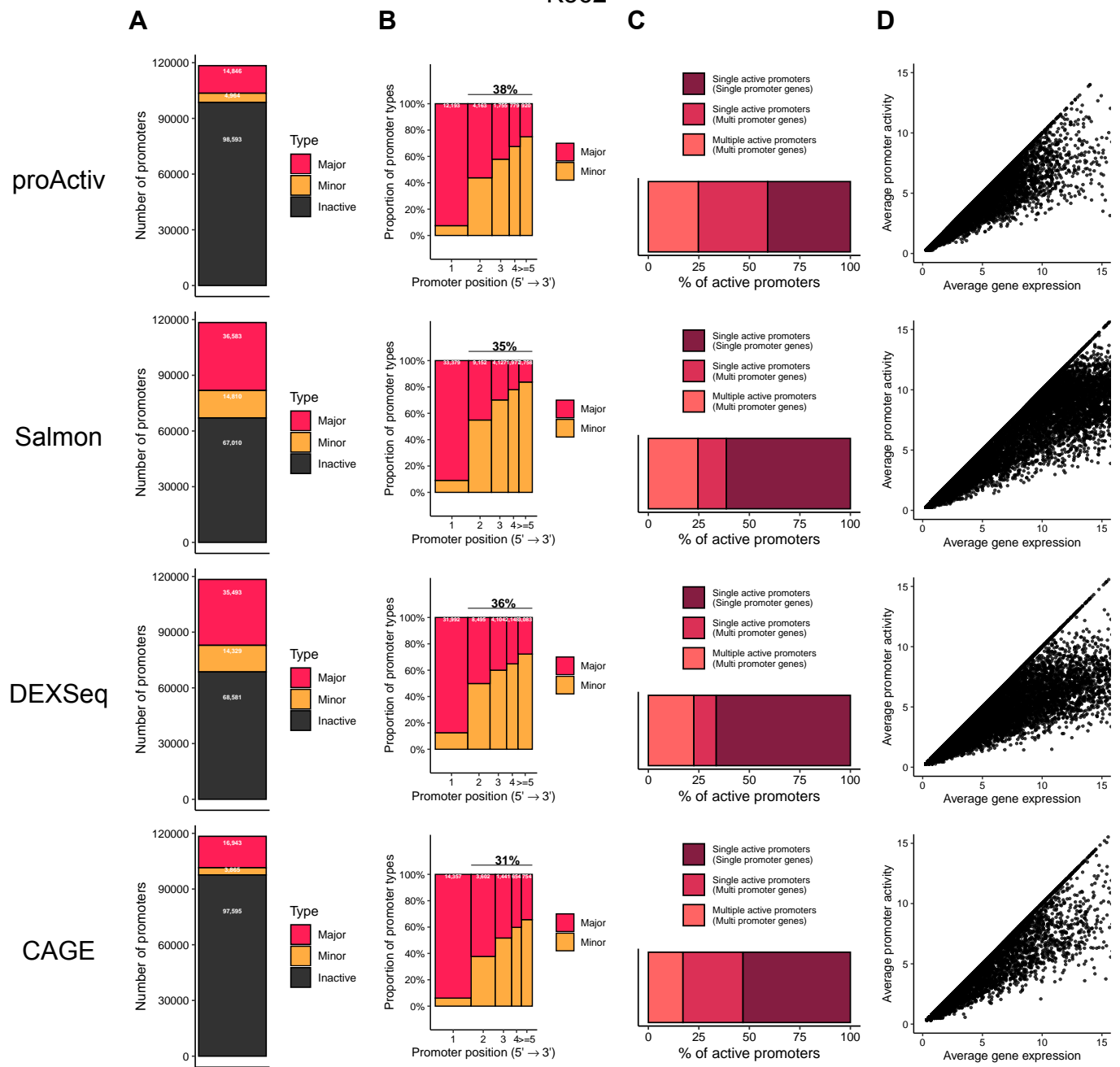

Supplement: Supplementary 1 — Figs. S1 to S10 Tables S1 to S5 [file csbj.0033.f1.zip › Supplemental Figure 3.pdf]

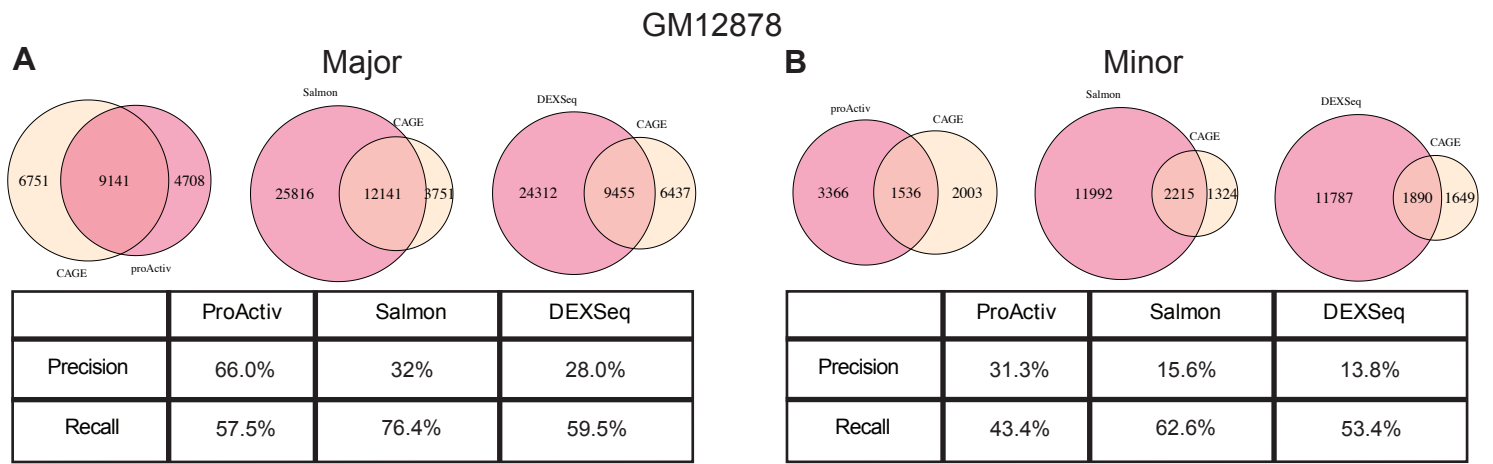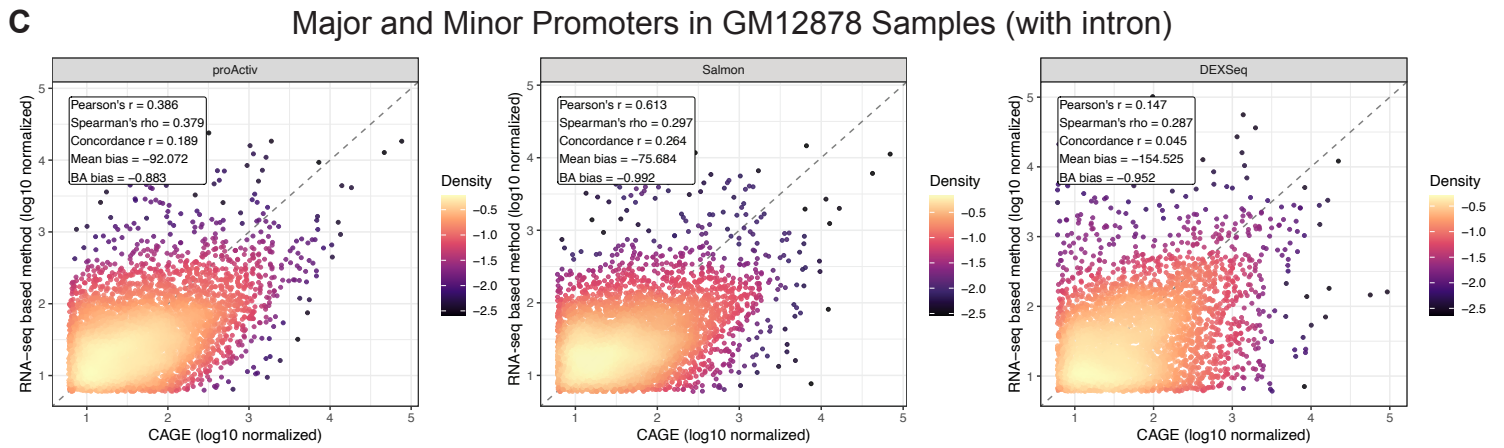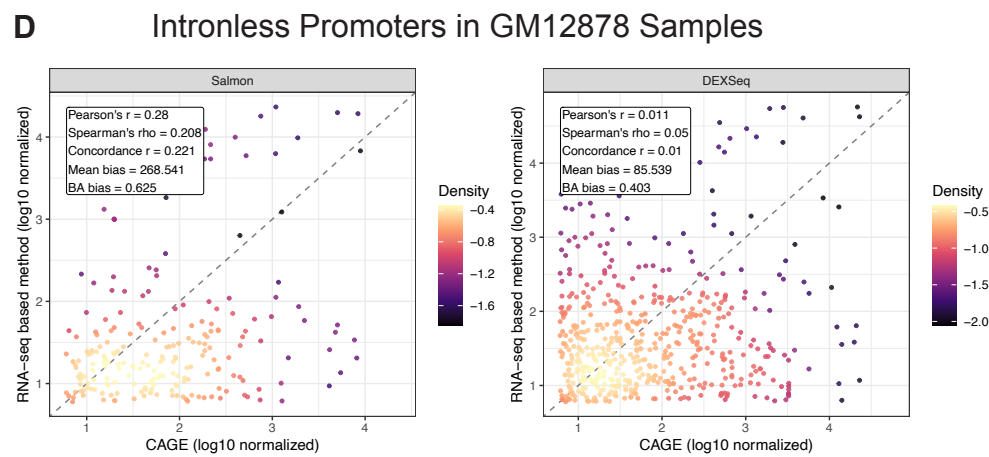

Supplement: Supplementary 1 — Figs. S1 to S10 Tables S1 to S5 [file csbj.0033.f1.zip › Supplemental Figure 5.pdf]

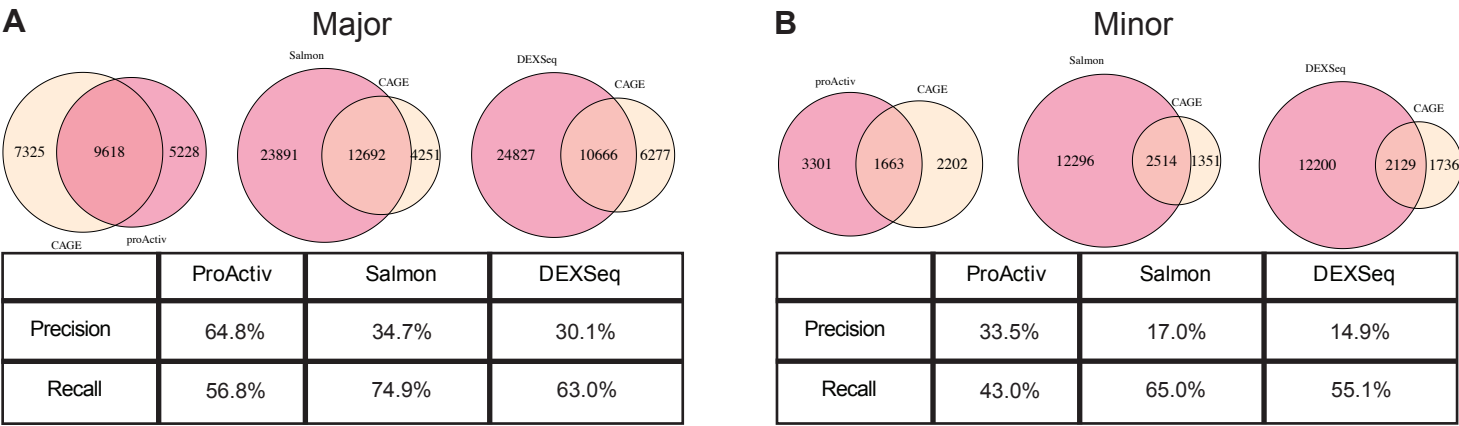

**C Major and Minor Promoters in K562 Samples (with intron)**

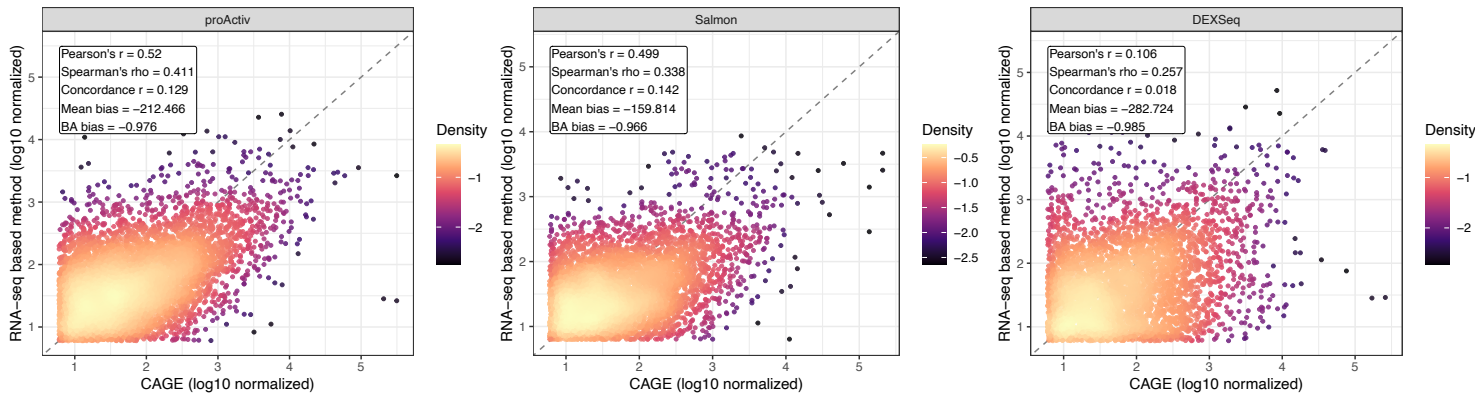

**D Intronless Promoters in K562 Samples**

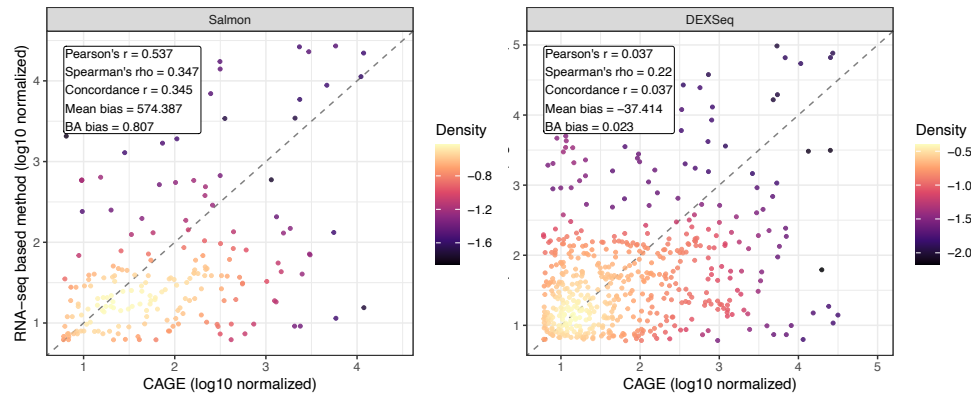

Supplement: Supplementary 1 — Figs. S1 to S10 Tables S1 to S5 [file csbj.0033.f1.zip › Supplemental Figure 6.pdf]

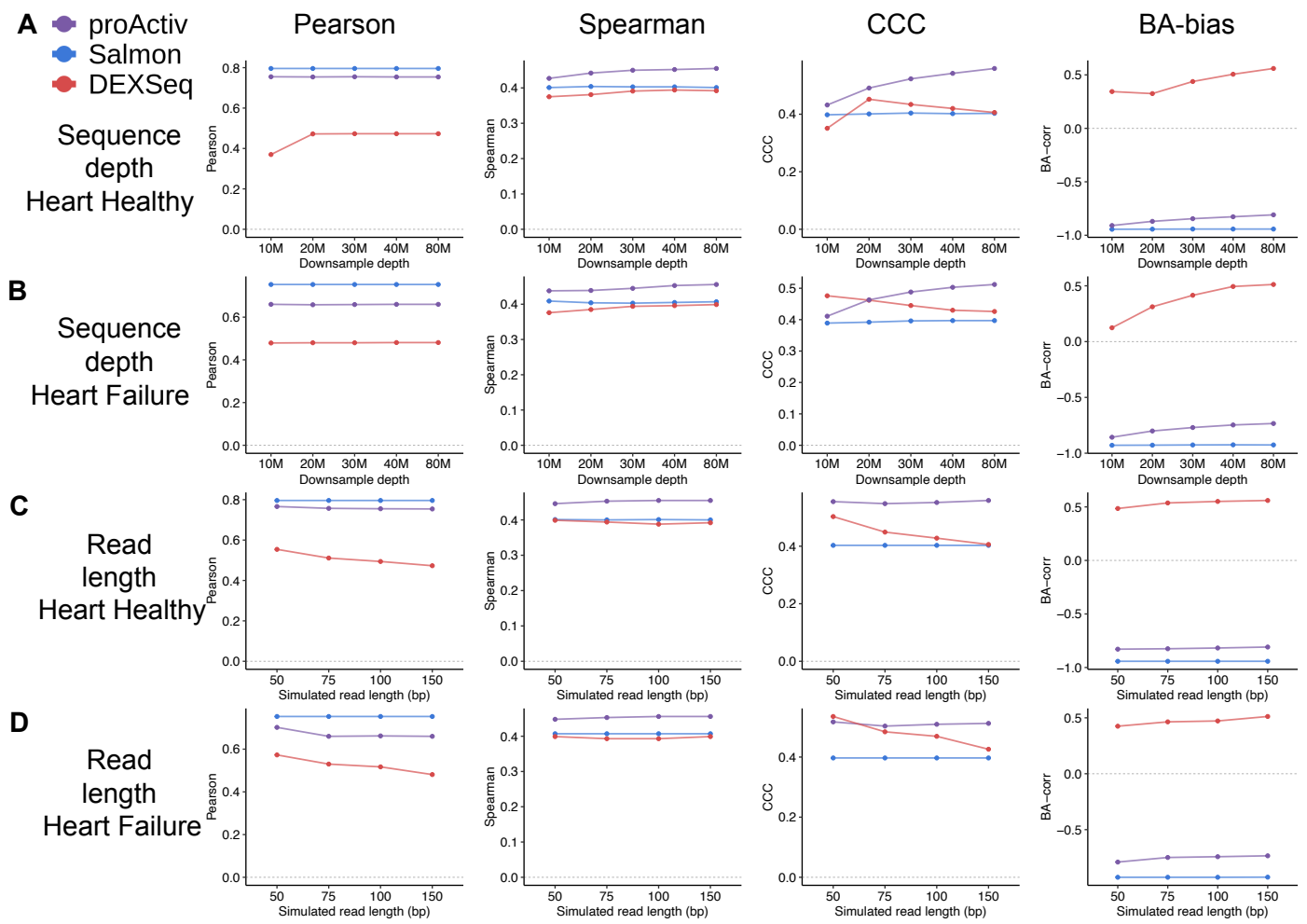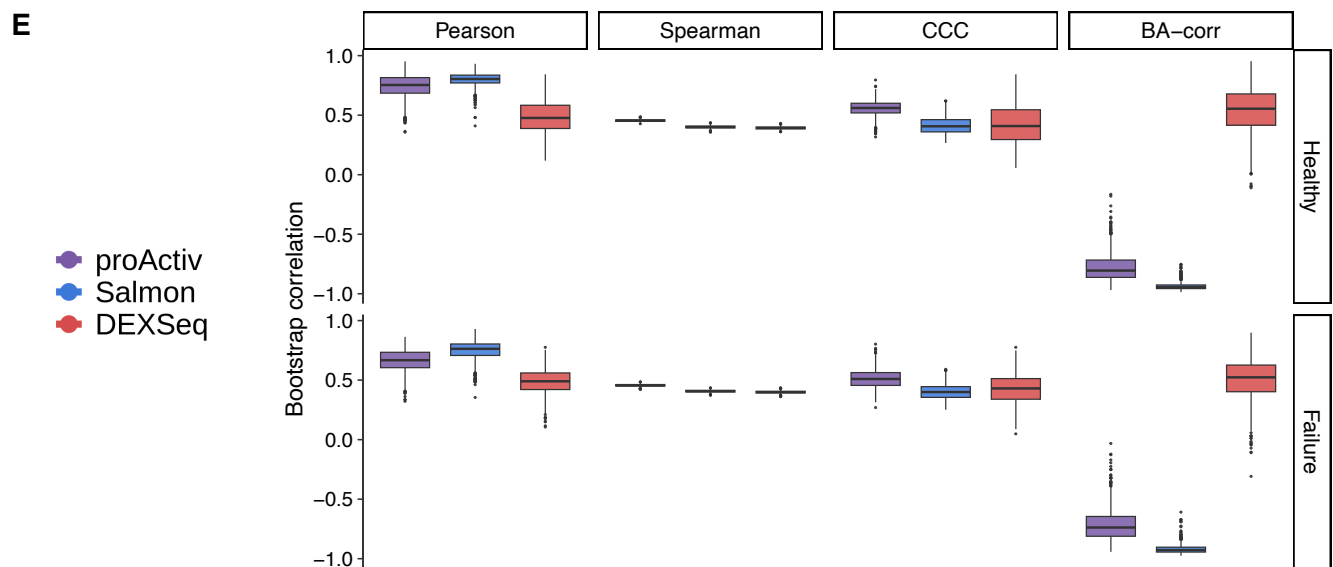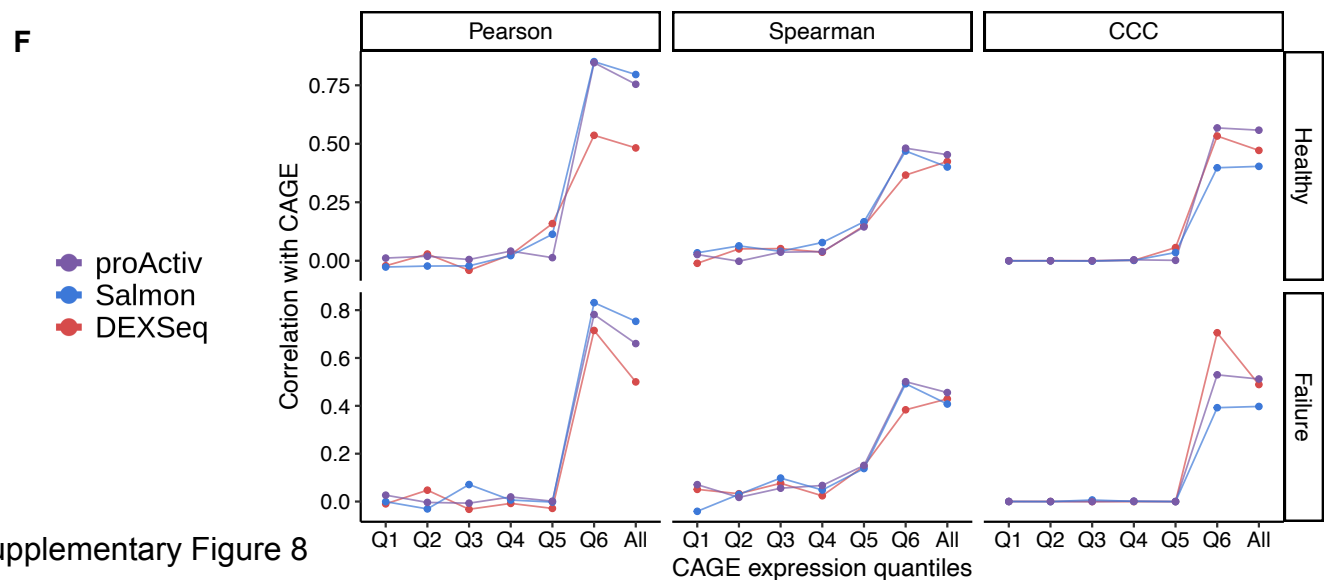

Supplementary Figure 8

Supplement: Supplementary 1 — Figs. S1 to S10 Tables S1 to S5 [file csbj.0033.f1.zip › Supplemental Figure 8.pdf]

A

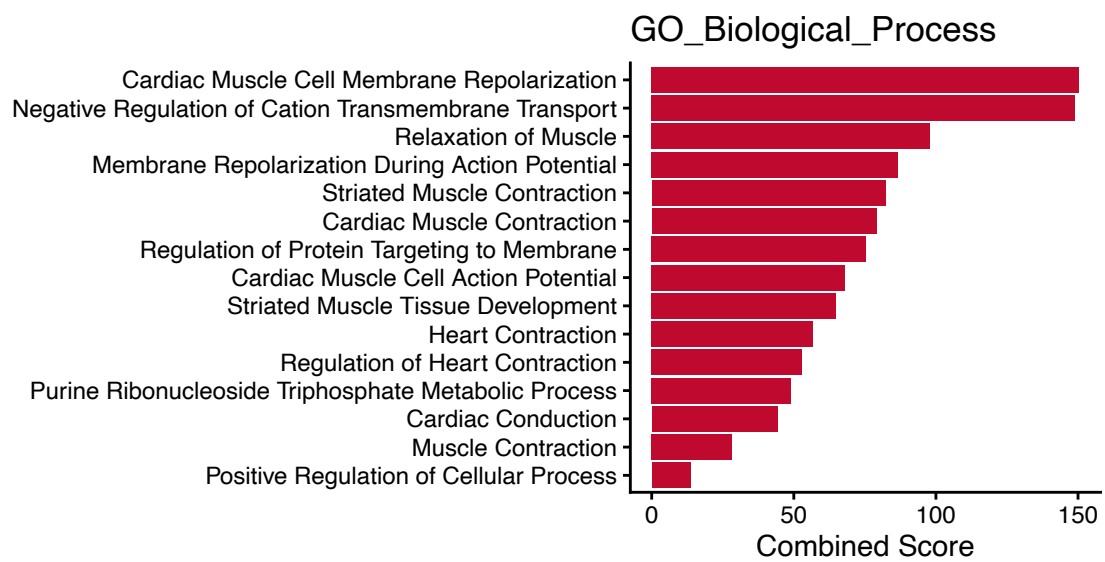

Supplement: Supplementary 1 — Figs. S1 to S10 Tables S1 to S5 [file csbj.0033.f1.zip › Supplemental Figure 9.pdf]
