# Supplementary material for: SnakeAltPromoter Facilitates Differential Alternative Promoter Analysis
Source: Comput Struct Biotechnol J. 2026 Apr 9;35(1):0033. doi: 10.34133/csbj.0033 (PMC13082578; doi:10.34133/csbj.0033)
Supplement: Supplementary 1 — Figs. S1 to S10 Tables S1 to S5 [file Supplemental Figure 7.pdf › Supplemental Figure 7.pdf]

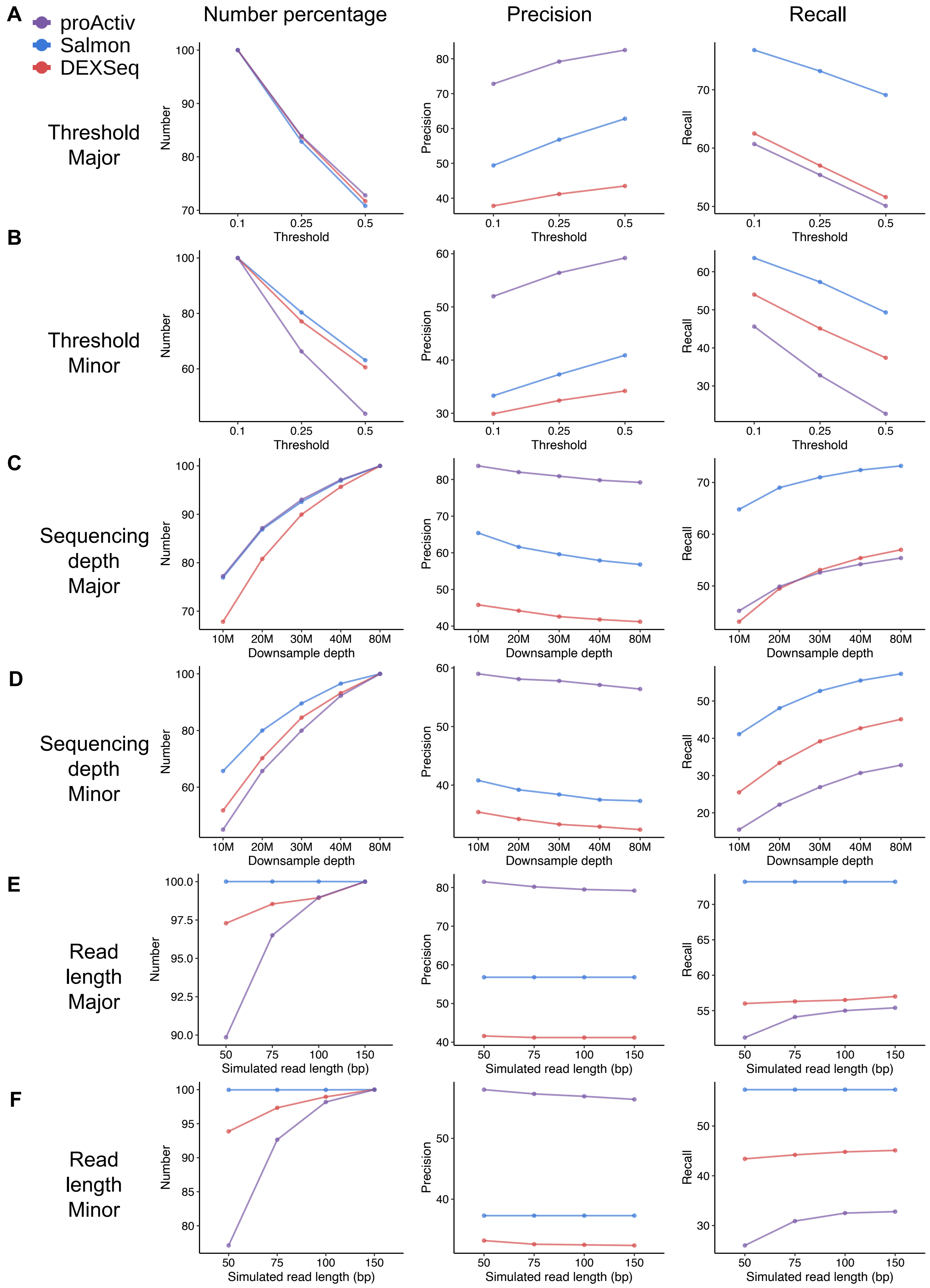

Supplementary Figure 7
